# Cardiovascular health in the menopause transition: a longitudinal study of up to 3892 women with up to four repeated measures of risk factors

**DOI:** 10.1186/s12916-022-02454-6

**Published:** 2022-08-17

**Authors:** Gemma L. Clayton, Ana Gonçalves Soares, Fanny Kilpi, Abigail Fraser, Paul Welsh, Naveed Sattar, Scott M. Nelson, Kate Tilling, Deborah A. Lawlor

**Affiliations:** 1 MRC Integrative Epidemiology Unit at the University of Bristol, Bristol, UK; 2 Population Health Sciences, Bristol Medical School, Bristol, UK; 3 Bristol NIHR Biomedical Research Centre at University Hospitals Bristol NHS Foundation Trust and University of Bristol, Bristol, UK; 4 Institute of Cardiovascular and Medical Sciences, University of Glasgow, Glasgow, UK; 5 School of Medicine, Dentistry and Nursing, University of Glasgow, Glasgow, UK

**Keywords:** ALSPAC, Cardiovascular, Menopause, Menopausal transition, Reproductive age, Time to final menstrual period, CIMT

## Abstract

**Background:**

Women experience adverse changes in cardiovascular health in mid-life; whether the menopausal transition influences these remains strongly debated. The aim of this study was to examine associations of reproductive age (time since final menstrual period (FMP)) with change in carotid intima media thickness (CIMT) and cardiovascular risk factors and determine the role of chronological and reproductive age.

**Methods:**

We used data from 1702 women from a pregnancy-based UK cohort who had up to four repeat cardiovascular health measures between mean age 51 (SD = 4.0) and 56 (SD = 3.6) years and experienced a natural menopause. Multilevel models were used to assess the relationship between cardiovascular measures and time since FMP (reproductive age), whilst adjusting for the underlying effects of chronological age and confounders (socioeconomic factors, body mass index, smoking, alcohol, parity, age at menarche). In addition, we looked at the relationship between cardiovascular measures by chronological age according to menopausal stages (pre-menopause, peri-menopause and post-menopause) using information from women who had and had not experienced menopause (*N* = 3892).

**Results:**

There was no strong evidence that reproductive age was associated with CIMT (difference in mean 0.8 μm/year, 95% CI − 0.4, 2.1), whereas there was a strong positive association of chronological age (7.6 μm/year, 95% CI 6.3, 8.9). Consistent with this, we found weaker linear associations of reproductive compared with chronological age for atherosclerotic risk factors, such as with systolic blood pressure (− 0.1 mmHg/year, 95% CI − 0.3, 0.1, and 0.4 mmHg/year, 95% CI 0.2, 0.5, respectively) and non-HDL-cholesterol (0.02 mmol/l/year, 95% CI 0.005, 0.03, and 0.06, 95% CI 0.04, 0.07, respectively). In contrast, associations with fat mass (0.06 kg/m^2^/year, 95% CI 0.03, 0.10, and 0 kg/m^2^/year, 95% CI − 0.04, 0.04, respectively) and C-reactive protein (0.01, 95% CI 0.001, 0.02, and 0.01, 95% CI − 0.001, 0.02 natural logged mg/l/year, respectively) were stronger for reproductive compared with chronological age. Both reproductive and chronological age were (weakly) positively associated with glucose (0.002, 95% CI 0.0001, 0.003, and 0.002, 95% CI 0.0001, 0.003 natural logged mmol/l/year, respectively).

**Conclusions:**

Our results suggest that going through the menopausal transition does not further increase women’s risk of atherosclerosis (measured by CIMT) beyond effects of ageing. Menopausal transition may, in additional to ageing, modestly increase adiposity and glucose levels and therefore a possible associated diabetes risk.

**Supplementary Information:**

The online version contains supplementary material available at 10.1186/s12916-022-02454-6.

## Background

Women experience adverse changes in cardiovascular health in mid-life, which may be influenced by the menopausal transition [1, 2]. There is evidence that these associations are driven, to at least some extent, by the hormonal changes that occur around the time of the menopausal transition [3]. However, much of the evidence comes from cross-sectional studies, where it is not possible to distinguish reproductive ageing (i.e. centred around the date of the final menstrual period (FMP)) from chronological ageing [4, 5], and hence whether menopause accelerates atherosclerosis remains controversial, even if it is commonly assumed to do so.

There is some evidence from cross-sectional and longitudinal studies (i.e. with repeat measurements across midlife) suggesting that menopause increases the risk of multiple cardiovascular risk factors, including increases in systolic blood pressure (SBP) [6, 7], hypertension, lipids [8–12], fasting glucose, diabetes, and fat mass [13], whilst associated with decreases in lean mass, in analyses adjusting for chronological age and several potential confounders [14]. Furthermore, most of these longitudinal studies have small sample sizes (most fewer than 500, with only four larger than 1000 [8, 12, 15, 16] which are sub studies of the larger Study of Women’s Health Across the Nation (SWAN) cohort). Once someone is diagnosed with a cardiovascular disease (CVD), they are considered ‘diseased’ (i.e. a status that cannot change further) and such diagnoses around the time of the menopause are rare, meaning that it is not possible to measure change in disease status across the menopause transition. However, sub-clinical measures of atherosclerosis, such as carotid intima media thickness (CIMT), a valid sub-clinical measure approved for use in randomised controlled trials (RCTs) [17], could provide valuable insights into the role of reproductive ageing and associated hormonal changes on disease risk. We were only able to identify two longitudinal studies exploring this, which showed increases in CIMT related to the menopause, independent of age at baseline, in 249 women with up to five repeat measures [18] and a larger study of 890 women [16].

The aims of this study were to (1) examine relationships of reproductive and chronological age with change in CIMT and multiple measures of cardiovascular health across mid-life in women; (2) identify whether change in cardiovascular risk factors across mid-life mediate any potential relationships of reproductive and chronological age with CIMT; and (3) examine the relationship between cardiovascular measures and chronological age according to menopausal stages (pre-menopause, peri-menopause and post-menopause) using information from women who had and had not experienced menopause.

## Methods

### Participants

Data from the mothers of the Avon Longitudinal Study of Parents and Children (ALSPAC) were used. ALSPAC invited all pregnant women resident in the area surrounding the city of Bristol, United Kingdom (UK), who had an estimated delivery date between 1 April 1991 and 31 December 1992 to the study, with approximately 15,454 pregnancies recruited to the study [19]. Full details of recruitment, follow-up, and data collection for these women have been reported elsewhere [19–21], and the study website contains details of all the data that is available through a fully searchable data dictionary and variable search tool (http://www.bristol.ac.uk/alspac/researchers/our-data/). Ethical approval for the ALSPAC Study was obtained from the ALSPAC Law and Ethics Committee and UK National Health Service Research Ethics Committees. Approximately 18 years after enrolment in ALSPAC (where enrolment was during the ‘index’ pregnancy), all mothers still engaged with ALSPAC (*N* = 11,264) were invited to join this study, which included detailed clinic assessments repeated up to four times [11, 19]. The clinic assessments were completed as follows: first, between 2009 and 2011 when 4831 women attended (median age 48, interquartile range [IQR] 45, 51); second, about 2.5 years later, between 2011 and 2013 when 2892 women attended (aged 51 [48, 54]); third, approximately 1.3 years later, in 2013–2014 when 3005 women attended (52 [49, 55]); and fourth, about 1 year later, in 2014–2015, when 2907 women attended (aged 53 [50, 56]).

**Fig. 1:**
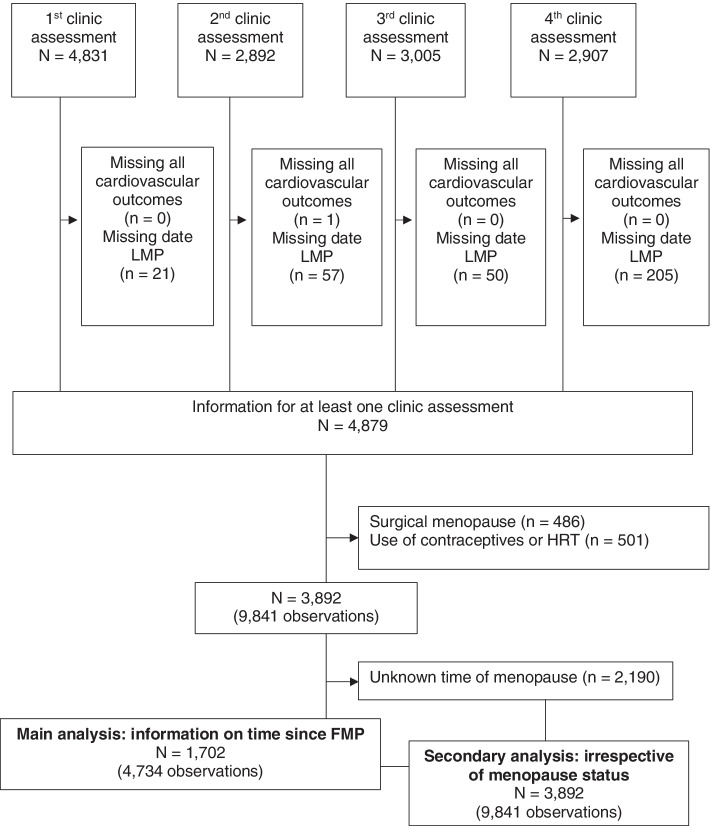
Participant flow into eligible and analysis groups in ALSPAC women, 2011-2015. LMP, last menstrual period; HRT, hormone replacement therapy. Surgical menopause refers to any woman who had any of the following: hysterectomy, oophorectomy, endometrial ablation, or radio- or chemotherapy related to reproductive organs

As we were interested in changes in cardiovascular health related to a natural menopausal transition (our target population), we excluded women who had experienced any of the following: hysterectomy, oophorectomy, endometrial ablation, or radio- or chemotherapy related to reproductive organs. Observations from women reporting using hormonal contraception or hormone replacement therapy (HRT) were censored at the measurement before starting hormones. Our study is based on women who attended at least one of these clinics, had data on cardiovascular factors and had experienced the menopause (at least 12 months with no menstrual periods) by the last clinical assessment (and therefore had information on time since their FMP, *N* = 1702 women with 4734 measures). In addition, we included women irrespective of whether they had gone through the menopause (because some women did not have 12 months of follow-up data after their final visit) and classified as pre, peri- or post-menopausal using the Stages of Reproductive Aging Workshop (STRAW) criteria, described in more detail below (*N* = 3892 women with 9841 measures). The participant flow for the main analyses is described in Fig. 1. The median time from first to last follow-up was 4.0 years (IQR: 0–4.8) in the whole sample [and 4.6 years (IQR: 4–5) when excluding those with only one time point]. When restricting to those who had a known date of menopause, the median time from first to last follow-up was 4.3 years (IQR: 1.6–5.0) and additionally those with CIMT data at the first and last clinic was 4.9 years (IQR: 4.6–5.3).

### Assessments of cardiovascular health

Change in CIMT was our primary outcome. This was measured at the first and last clinic assessments only as we did not anticipate substantial change in women of this age over the 12–18 months’ time between the middle clinics. Both the left and right common carotid artery scans were obtained via high-resolution B ultrasound and imaged longitudinally 1 cm proximal to the carotid bifurcation following a standardised protocol using a ZONARE z.one Ultra convertible ultrasound system with L10-5 linear transducer at the first assessment and a portable ultrasound scanner CardioHealth® Station at the last assessment. Ten-second cine loops were recorded in DICOM format and analysed offline using Carotid Analyzer for Research (Vascular Research Tools 5, Medical Imaging Applications, LLC 2008). Three consecutive cardiac cycles were identified and three measures of CIMT were taken from end-diastolic frames and averaged for both right and left carotid arteries. The mean of the left- and right-sided readings was used in analyses. The images were analysed by a single trained reader. All other cardiovascular measures were collected at each of the four clinic assessments.

Weight and height were measured according to standard protocols, with women wearing light clothes and no shoes. Weight was measured using an electronic scale with accuracy of 100 g (Tanita TBF-401) and height was measured using a Harpenden stadiometer and recorded to the nearest 1 mm. Body mass index (BMI) was then calculated by dividing weight (kg) by height squared (m^2^). Total fat and lean mass were derived from whole body dual energy X-ray absorptiometry (DXA) scans using a Lunar prodigy narrow fan beam densitometer. These were height-adjusted by dividing by height squared in the analyses.

SBP and diastolic blood pressure (DBP) (recorded in mmHg) and pulse rate (bpm) were measured using an Omron M6 upper arm BP/Pulse monitor. Blood pressure and pulse rate were measured twice in each arm, and the average of these measurements across arms was derived for each time point.

Blood samples were taken following standardised protocol. Participants were instructed to fast overnight or for at least 6 h prior to their clinic visit. After collection, the blood was immediately centrifuged and frozen at − 80 °C. The samples were assayed within 3 years of storage with no previous freeze–thawing cycles.

Fasting triglycerides, high-density lipoprotein cholesterol (HDL-c), total cholesterol (mmol/l), fasting glucose, and C-reactive protein (CRP, mg/l) were measured using an automated biochemistry analyser and using the manufacturers’ calibration and quality control materials (c311, Roche Diagnostics, Burgess Hill, UK). Non-HDL-c was derived by taking HDL-c away from total cholesterol.

### Reproductive and chronological age and menopausal status

Women were asked a detailed set of questions about their menstrual periods at each clinic assessment. FMP was identified when at least 12 months of amenorrhea had occurred since the date of the last menstrual period. Using this information, for each woman, reproductive age was calculated in years before and after the date of their menopause; hence, 0 is the date of their FMP, − 1 would be one year before that date, and + 1, one year after that date [22]. For women irrespective of menopausal status, the STRAW criteria were used to categorise them into one of three mutually exclusive reproductive stages at each assessment: (i) pre-menopausal (late reproductive age, STRAW category -3b and -3a); (ii) menopausal transition (peri-menopause), STRAW category -2 and -1; and (iii) postmenopausal, STRAW category ≥ 1a (irrespective of the years since menopause) [11]. Chronological age was self-reported at each clinic assessment. Information on use of hormonal replacement and contraception (used to censor follow-up in the analyses) and hysterectomy, oophorectomy, endometrial ablation, or radio- or chemotherapy related to reproductive organs (used to exclude women unable to experience a natural menopause) were also obtained from self-completed questionnaires at each assessment.

### Confounders

Confounders were defined *a priori* based on them being known or plausible causal factors for age at menopausal transition and cardiovascular risk [23]. We adjusted analyses for the following observed confounders (all were based on self-report): age at menarche (early (≤ 11 years), average (12–14 years), late (≥ 15 years)), BMI (kg/m^2^), smoking (never, past, current), parity (1, 2, 3, ≥ 4 pregnancies), alcohol (never or ≤ 4 times/month, 2–3 times/week, ≥ 4 times/week) and maternal education. Maternal education was defined by the highest attained qualification (i) Certificate of Secondary Education (CSE), ordinary- (O-level or vocational certificate (qualifications usually obtained at age 16, the UK minimum school leaving age when these women were at school), (ii) Advanced A-level (usually taken at 18 years); or (iii) university degree (Table 1). Smoking status and alcohol intake were self-reported at, or before, the first clinic. Information on self-reported age at menarche, pre-pregnancy BMI, number of previous pregnancies and maternal education were obtained around the time of recruitment to the study (mean age 28.3 years, SD 4.8), with information from subsequent questionnaires used to update parity (last data obtained at similar time of the first mid-life clinic assessment when women were mean 48.4 (SD 4.4) years old).

**Table 1:** Distribution of potential confounders (*N* = 1702)

	*N* (%)
Pre-pregnancy BMI	
Normal	1287/1496 (86.0%)
Overweight	170/1496 (11.4%)
Obese	39/1496 (2.6%)
Smoking status (obtained at, or before, the first clinic)	
Never smoker	809/1483 (54.6%)
Former smoker	525/1483 (35.4%)
Current smoker	149/1483 (10.0%)
Alcohol intake frequency (obtained at, or before, the first clinic)	
Never or less than 4 times a month	468/1155 (40.5%)
2 to 3 times a week	384/1155 (33.2%)
4 or more times a week	303/1155 (26.2%)
Parity	
1	193/1702 (11.3%)
2	567/1702 (33.3%)
3	423/1702 (24.9%)
4+	518/1702 (30.5%)
Age at menarche (taken at time of recruitment to the study (mean age 28.3, SD 4.8))	
Early (≤ 11 years)	242/1507 (16.1%)
Average (12-14 years)	1055/1507 (70.0%)
Late (≥ 15 years)	210/1507 (13.9%)
Educational achievement (taken at time of recruitment to the study (mean age 28.3, SD 4.8))	
CSE/vocational degree/ O-level	688/1587 (43.4%)
A-level	498/1587 (31.4%)
University degree	401/1587 (25.3%)

*A-level* advanced level, *BMI* body mass index, *CSE* certificate of secondary education, *O-level* ordinary level. In models pre-pregnancy BMI is a continuous variable

### Statistical analyses

We used multilevel models to examine associations of each cardiovascular health outcome with chronological and reproductive age, allowing for repeated measures within women. The multilevel models include all women with at least one cardiovascular measure, under the missing at random (MAR) assumption. We used fractional polynomials to allow the relationship between cardiovascular outcomes and chronological age and reproductive age to be non-linear, and linear models if no evidence of non-linearity was found (*p*-value for evidence at 5% significance level). Linear and non-linear models were compared using Akaike Information Criterion (AIC) and Bayesian Information Criterion (BIC). When determining whether the relationship was non-linear or not, we restricted the data at the 5th and 95th centiles of reproductive or chronological age to prevent overfitting in the tails of the distribution. Cardiovascular health outcomes were modelled against reproductive age alone, chronological age alone and with both time scales in the model. Model fit was assessed using AIC and BIC. We present the model including both reproductive and chronological age, mutually adjusted for each other and for all known potential confounders. All models were fit with a random intercept for each woman and random slope for chronological age to allow chronological age to have a different effect on the outcome for each woman. In the univariable models assessing the relationship between reproductive age and our cardiovascular outcomes, a random slope for reproductive age was instead included. Additional file: Table S1 details characteristics of the model fit.

Our main analyses where time since FMP was the key exposure can only include women who are known to have gone through the menopause, as this is required to calculate reproductive age. This might introduce selection bias, and to explore this, we compared relationships in cardiovascular outcomes by chronological age according to menopausal stages (pre-menopause, peri-menopause and post-menopause) in all women, irrespective of whether or not they had gone through the menopause (*N* = 3892 women with 9841 observations). If we see no association of reproductive age with an outcome but a positive linear association of chronological age, we would expect the association of chronological age with that outcome to have a similar slope across all three menopausal status strata. By contrast, if there was a strong positive association with change in an outcome across reproductive age but little or no association with chronological age, we would expect to see little association of chronological age with the outcome in the pre-menopausal strata, little or a weak positive slope in the peri-menopausal strata and a stronger positive slope in the post-menopausal strata. Departure from these expectations could be due to selection bias.

For cardiovascular factors where we saw evidence of a change across reproductive or chronological age, we explored the extent to which they may mediate differences in CIMT between the first and the last data collection. Mediation was assessed with the cardiovascular health measures at clinic assessments 2 and 3 explored as potential mediators [24, 25]. These timepoints were used because they occur after the first CIMT and before the second CIMT measure and hence could be plausibly influenced by the first and influence the last CIMT measures. We explored mediation using measures at clinic 2, clinic 3, and the difference in the measures between 2 and 3, separately. The total effect of reproductive or chronological age (at the first clinic assessment) on CIMT (at the last clinic assessment) was estimated by regressing CIMT on reproductive or chronological age, adjusted for chronological or reproductive age respectively, CIMT (at the first clinic assessment), and baseline confounders. The direct effect (i.e. effect of reproductive or chronological age on CIMT not via the mediator) was estimated in the same way as the total effect but additionally included the mediator. The mediator was also regressed on reproductive or chronological age, adjusted for chronological or reproductive age CIMT (at the first clinic assessment) and baseline confounders. The indirect effect (i.e. effect of reproductive or chronological age via the mediator) was estimated by multiplying the coefficient for age in this latter regression and the coefficient for the mediator in the previous regression model. Standard errors were obtained via bootstrapping.

### Missing data

There was missing data on some of the cardiovascular health measures (< 5% of women who had information for at least one outcome at a clinic assessment, had missing information for at least one other outcome clinics) and confounders, in particular for alcohol (32%) and smoking (13%). These were imputed using multivariable multiple imputation with chained equations, performed using the *mi* *impute* command in Stata 17 [26]. We used 50 imputed data sets and included all variables included in any models (including all outcomes) in the imputation models. Data on smoking status and alcohol intake from questionnaires completed up to 7 years prior to the first mid-life clinic were also used in the prediction models for missing risk factors. The amount of missing data and the characteristics before and after imputation are presented in Additional file: Tables S2 and S3.

### Sensitivity analyses

To explore the sensitivity of our results to including all women who participated in at least one clinic assessment, we also repeated analyses only in women with three or four repeat measures (and both measures for CIMT). We compared our main analyses (using multivariable imputation for missing outcome and/or confounder values) to analyses including only those with complete data on confounders. We also repeated all analyses using general estimating equations (GEE) to check the robustness of the results, as the GEE assumptions are different to multilevel models. GEE assume missing completely at random (compared to MAR in multilevel models) and are more robust to the misspecification of the covariance structure of the random effects. We repeated our main analysis excluding any women who had plaque and therefore had their CIMT measured outside the area of plaque.

## Results

Women included in the analysis were aged 37-61 years at the first assessment and 43-66 years at the last follow-up. The median age of menopause in the sample was 49.5 years (IQR 47.3 to 51.8), with 50 (3%) experiencing premature ovarian insufficiency (POI) (menopause aged < 40 years) and 153 (9%) early menopause (menopause aged 40- < 45 years). The median age and IQR of women, in the whole sample (*N* = 3892), by pre, peri, and post-menopausal stages were 46 (44–49), 50 (49–52), and 55 (52–57), respectively. BMI, smoking status, alcohol intake, parity, age at menarche, and education categories at women’s first assessment are shown in Table 1. Overall, 86% of the women had a normal pre-pregnancy BMI, 55% were never smokers, 40% consumed no alcohol or consumed it less than 4 times/month, 11% had experienced only one pregnancy, 70% had age at menarche between 12 and 14 years, and 25% had university degree. Additional file 1: Table S4 shows distributions of menopausal stage, age, and cardiovascular outcomes in each clinic assessment and according to menopausal stages. 20 (1.2%) women had CIMT evidence of plaque or stenosis. The median CIMT was 0.56 mm (IQR: 0.52, 0.59) at clinic 1 and 0.60 (0.55, 0.67) at clinic 4. The rate of change in CIMT per chronological year was 0.008 mm [0.007, 0.009] (Additional file 2: Table S5).

**Fig. 2:**
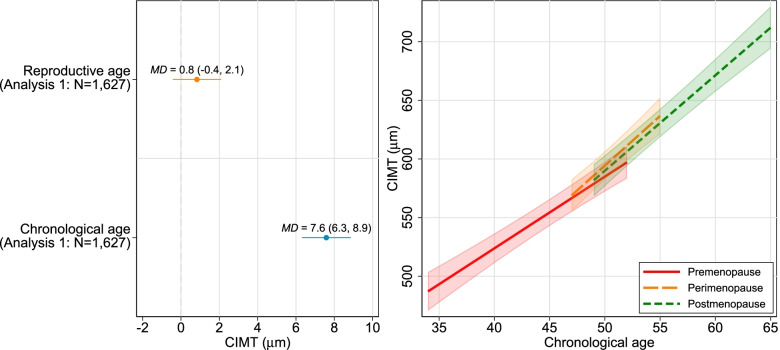
Associations of reproductive and chronological aging with carotid intima media thickness. The left-hand panel shows the main analysis results: mean difference in carotid intima media thickness (CIMT) per year for reproductive and chronological age with mutual adjustment for each age variable and pre-pregnancy body mass index (BMI), age at menarche, parity, maternal education, smoking status and alcohol intake, undertaken in 1627 women with 2561 observations. The right-hand panel shows the additional analyses undertaken to explore possible selection bias in the main analysis. The figure shows the mean difference in CIMT per year in chronological age by menopausal stage (pre, peri or post), undertaken in all eligible women (total *N* = 3765 women with 5628 observations, with 2238 (2652), 1209 (1254) and 1344 (1722) women (observations) contributing data to pre, peri and post-menopause, respectively). Each trajectory was restricted to the middle 95% of the data, except for the youngest ages in the premenopausal group and the oldest ages in the postmenopausal group. The 5th and 95th percentile of chronological age in pre, peri and post-menopausal stage are 40–52, 47–55, and 49–61, respectively. The *p*-value for interaction, a test for any difference in the linear association between any of the three menopause stages, is reported in Table S6 (and by age in Table S7)

**Fig. 3:**
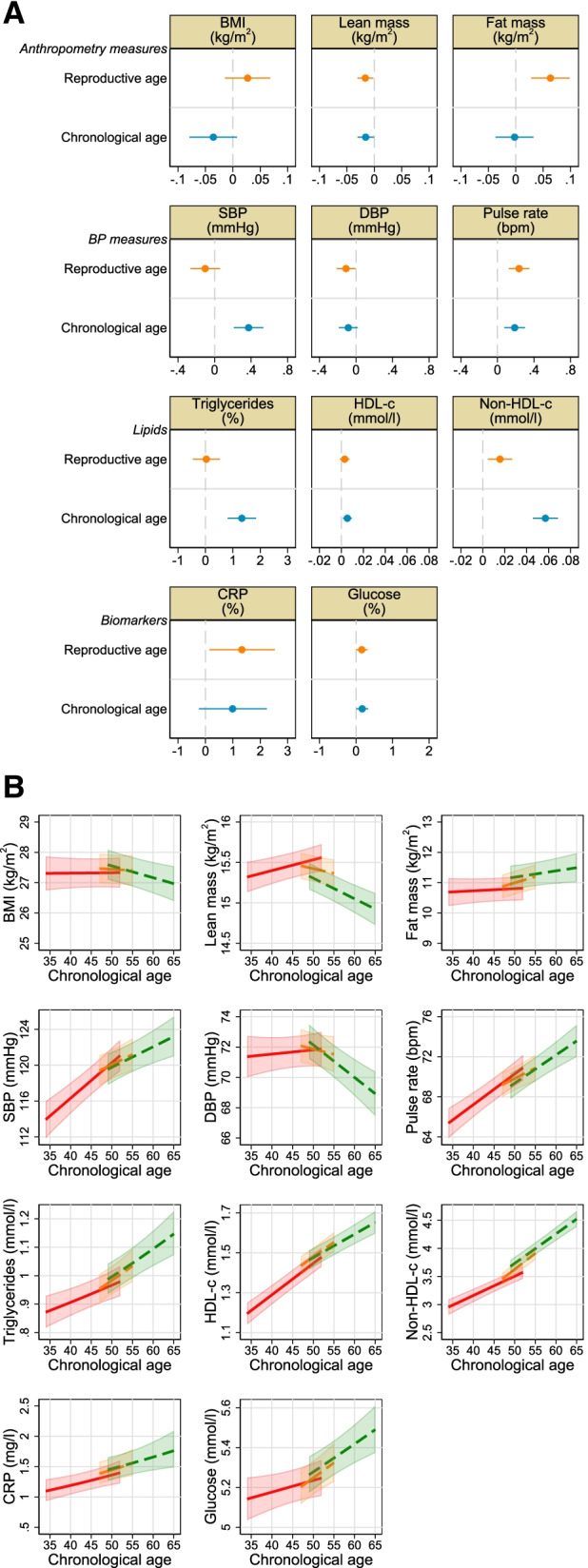
Associations of reproductive and chronological ageing with cardiovascular measures including anthropometry, blood pressure, lipids and C-reactive protein and glucose. Figure 3(**a**) shows the main analysis results: mean difference in cardiovascular measures per year for reproductive and chronological age with mutual adjustment for each age variable and pre-pregnancy body mass index (BMI), age at menarche, parity, maternal education, smoking status, and alcohol intake, undertaken in 1702 women with 4734 observations. The best fitting model between reproductive age and fat mass and non-HDL-c was reproductive age to the power of a half and the addition of a cubic term respectively; however, the linear term is shown here for completeness. The non-linear models are graphically shown in Supplementary Figure S1. The *p*-value for interaction, a test for any difference in the linear association between any of the three menopause stages, is reported in Table S6 (and by age in Table S7). Figure 3(**b**) shows the additional analyses undertaken to explore possible selection bias in the main analysis. This shows the mean difference in cardiovascular measures per year in chronological age by menopausal stage (pre, peri or post), undertaken in all eligible women (total *N* = 3892 women with 9841 observations, with 2313 (4118), 1666 (2388) and 1550 (3335) women (observations) contributing data to pre, peri, and post-menopause, respectively).

Most associations of both chronological and reproductive age with the cardiovascular outcomes were linear, and therefore, linear associations are presented as the main results. Additional file 2: Figure S1 shows non-linear associations for fat mass and non-HDL-c. Figures 2 and 3 show associations of reproductive and chronological age with CIMT and risk factors respectively, mutually adjusted for reproductive and chronological age and all measured confounders and the relationship between CIMT and risk factors respectively with chronological age according to menopausal stages (pre-menopause, peri-menopause and post-menopause) in women with known and unknown date of menopause (coefficients are presented in Additional file 2: Tables S6–S7). Results from other models (with both chronological and reproductive age unadjusted and mutually adjusted) are shown in Additional file 2: Figure S2 and Table S5.

We found no strong evidence that reproductive age influenced change in CIMT (0.8 μm/year, 95% CI − 0.4 to 2.1), whereas there was a positive association with chronological age (7.6 μm/year, 95% CI 6.3 to 8.9) in the equivalent analyses (Fig. 2). Results stratified by menopausal status using all women, irrespective of whether date of menopause was known or unknown, were consistent with our main analysis, showing similar positive associations of chronological age with CIMT in all three menopausal stages (Fig. 2; Additional file 2: Tables S6–S7). Although there was statistical evidence to show a change in CIMT/year by menopause status (Fig. 2, Additional file 2: Table S6), this difference between the groups appears to be small and the overall the change is linear, suggesting very little difference between the menopause status categories.

Associations of reproductive and chronological age with BMI were in opposite directions: reproductive age was associated with higher BMI, whilst chronological age was associated with lower BMI (Fig. 3(a)). This seems to be explained by results for the separate components of body weight. Reproductive (− 0.02 kg/m^2^/year, 95% CI − 0.03, − 0.002) and chronological (− 0.02 kg/m^2^/year, 95% CI − 0.03, − 0.001) age were associated with lower lean mass, whilst reproductive age was associated with higher fat mass (0.06 kg/m^2^/year, 95% CI 0.03, 0.10) and no association was observed for chronological age (0 kg/m^2^/year, 95% CI − 0.04 to 0.04). Stratified analyses were consistent with the main analyses, showing decreases in both BMI and lean mass with chronological age in the post-menopause compared to no change (BMI) or a modest increase (lean mass) in pre-menopause, whereas for fat mass there was a greater increase with age in the peri- and post-menopausal periods than in pre-menopause (Fig. 3(b)).

We found weaker linear associations of reproductive compared with chronological age for SBP: − 0.1 mmHg/year (95% CI − 0.3, 0.1) and 0.4 mmHg/year (95% CI 0.2, 0.5), respectively. Reproductive and chronological age were negatively associated with DBP and positively associated with pulse rate.

We also found weaker linear associations of reproductive compared with chronological age for non-HDL-cholesterol (0.02 mmol/l/year, 95% CI 0.005, 0.03, and 0.05, 95% CI 0.04, 0.07, respectively). Whilst we found no evidence of an association of reproductive age with triglycerides, we found a positive association with chronological age.

Both reproductive and chronological age were (weakly) positively associated with glucose (natural log scale: 0.002 mmol/l/year, 95% CI 0.0001, 0.003, and 0.002 mmol/l/year, 95% CI 0.0001, 0.003, respectively). Similarly, for CRP. Analyses stratified by menopausal status were also consistent.

We found no evidence to suggest that any of the risk factors potentially affected by chronological ageing (lean mass, SBP, DBP, pulse rate, triglycerides, non-HDL, and glucose) mediated the relationship between chronological age and CIMT (Additional file 2: Table S8). As we found no evidence for an effect of reproductive age on CIMT, exploring whether this was mediated by the risk factors was not relevant.

Results for reproductive and chronological age models were similar when only women with 3 or 4 repeated measures and those with complete information were analysed (Additional file 2: Figure S3). When removing 20 (1.2%) women with a plaque, our results did not change (Additional file 2: Figure S4).

## Discussion

### Main findings

Our results suggest that reproductive age (reflecting the menopausal transition) does not independently influence change in sub-clinical atherosclerosis (CIMT) or risk factors (e.g. SBP, non-HDL-cholesterol and triglycerides) strongly associated with atherosclerosis, as shown in randomised trials and/or Mendelian randomisation studies to causally influence coronary heart disease [17, 27, 28]. By contrast reproductive age may increase adiposity and risk of diabetes, albeit modestly, as suggested by stronger positive linear associations with reproductive age than chronological age for BMI, fat mass, and fasting glucose. HRT may not identically reflect endogenous hormonal and other changes associated with a natural menopause. However, it is notable that our findings have some consistency with randomised controlled trials of HRT, which have shown no protection, or a possible increased risk for coronary heart disease and reduced risk for type 2 diabetes [29].

### Strengths and limitations

To our knowledge, this is the largest prospective study to date with two repeat CIMT measures and up to four repeated cardiovascular risk factor measures that spans the late reproductive period, from menopausal transition into post menopause. The average 5-year follow-up period with up to four repeat measures in women of different baseline ages allowed the description of associations from 4 years before to 16 years after the menopause, a longer postmenopausal period than described in previous studies.

We used multilevel models, which allow all women with at least one measurement occasion to be included in the analysis under the MAR assumption, i.e. missingness depends on observed data and therefore associations do not differ in women (with the same characteristics) who have fewer repeat measures. Furthermore, sensitivity analysis restricted to women who had three or four repeat measures showed similar results to those with at least one repeat measure. We had to restrict our main analyses to women in whom we could calculate their FMP, meaning only those who has at least 12 months since their last period could be included. This could introduce selection bias. However, consistency of our main analysis findings with those of the associations of change in outcome with chronological age by strata of menopausal status suggests our findings are not substantially biased by selection. We do however note that a woman’s menopausal stage will reflect her chronological age (i.e. at the time of baseline assessment, women who are pre-menopausal will be on average younger than those who are postmenopausal). We therefore need to be cautious in our interpretation and in the magnitude of associations which are likely to be driven by differences in the age distribution across the groups.

We fit models which included time since FMP and chronological age to separate the influence of both chronological and reproductive age. However, given that chronological age is the sum of age at menopause and time since FMP, we could have instead analysed time since FMP and age at menopause only, a reparameterisation of time since FMP and chronological age. As such, in mutually adjusted models, the coefficients of chronological age are equivalent to that of age at menopause, whilst time since FMP in the model including age at menopause is the sum of time since FMP in the model including chronological age and additionally the coefficient of chronological age.

As reproductive age is a self-reported measure and measured with more error than chronological age, it may be that this causes some bias towards the null for reproductive age, and correspondingly away from the null for chronological age.

Distributions of outcomes and confounders were similar between women included and excluded from the main analysis (Additional file 3: Tables S9–S10).

Our study is predominantly of White European origin women, and previous studies have shown ethnic differences in cardiovascular risk factors [30], so our findings might not be generalisable to women of other race/ethnic groups. As our study recruited women during an index pregnancy and only followed those with a live birth from that pregnancy, all participants had at least one live birth and we cannot assume that our findings would generalise to women with no previous pregnancies or live births. As we know the risk of cardiovascular disease increases with an increase in live births [31], the association between reproductive age and cardiovascular health may differ in studies that also include nulliparous women. Vasomotor symptom severity and duration are also known to associate with HRT use (the most effective treatment for these symptoms) and CVD risk. Censoring those who use exogenous hormones because we could not determine age at a natural menopause could induce some collider bias [32] if there is residual confounding between HRT and CVD. However, given the key confounders of HRT-CVD effects are the same as those for time to FMP and CVD (e.g. age, BMI, education) which we already adjust for, we anticipate that any bias would be small.

When restricting our sample to women with a time since FMP greater than 0, 71% of the sample, the median time (IQR) since FMP was 5.7 years (4.2–8.8). We believe this time is long enough to observe any differences in CVD risks possibly related to the menopause. However, it may be possible that the longer women are followed up after menopause, evidence of associations become apparent, or the observed associations become larger in magnitude. Furthermore, given only 12% (203/1702) of the sample experienced early menopause, it is possible that women at the very low end of the age at menopause distribution are indeed at increased risk and we were not able to pick this up. These analyses are in unselected women in mid-life and only 20 (1.2%) had evidence of plaque or atherosclerosis, highlighting the need for further follow-up into older ages.

### Comparisons with other longitudinal studies

We were able to identify ten papers published up to December 2021 that either explored change in cardiovascular outcomes by reproductive age [8, 12, 15, 16, 33–35] or change with chronological age within strata of menopausal status [18, 36, 37]. We have summarised these in Additional file 4: Table S11 [8, 12, 15, 16, 18, 33–37] including number of women, number of repeated measures, sample characteristics and key results. With one exception, these included fewer than 500 women [18, 36, 37]. The one exception was the SWAN which included between 249 to 2659 women in different publications [8, 12, 15, 16, 18, 33, 35].

Only two of these explored associations with CIMT [16, 18]. El Khoudary et al. [18] included 249 participants, (122 premenopausal, 115 early peri-menopausal, 4 late peri-menopausal and 8 postmenopausal at baseline) and in line with our results found that CIMT increased in post-menopause (0.024 mm/year, *p*-value 0.03) compared to pre-menopause, adjusting for age at baseline and ethnicity. Similarly, the recent SWAN paper [16] included 890 women with CIMT measures and suggested that older age at menopause was associated with an increase in CIMT.

Consistent with our results, Greendale et al., in a sub study of SWAN with *N* = 1246 [15], found an independent association between reproductive ageing and gain in fat mass and loss of lean mass until 2 years after the FMP in women who had an average age at FMP of 52 years. Our findings, with larger numbers, add to this evidence in suggesting that reproductive age, independent of chronological age, increases body fat.

Unlike our findings, Derby et al. [8] found increases in triglycerides with reproductive age, having adjusted for chronological age; however, this change was small. As in our study, Matthews et al. [12] found increases in triglycerides in midlife were small and largely related to chronological age rather than reproductive age or menopausal status. A weak positive linear change in non-HDL-c with reproductive age, consistent with our results, was also shown in that study. In a previous analysis of the same cohort (ALSPAC) using a metabolomic and largely lipids platform, Wang et al. found important changes in many lipids across the menopausal transition, taking into account chronological age [11], however, data were available for only two time points.

Reproductive and chronological age were weakly positively associated with fasting glucose in our study whilst the SWAN studies found neither or a negative association [12, 33, 37]. However, our study was considerably larger than the others. Furthermore, the decrease with reproductive or chronological age would be surprising given in general populations diabetes increases with age [14].

Some studies have looked specifically at the association of early or premature menopause as a risk factor for CVD [38–40]. Daan et al. compared 83 women previously diagnosed with POI (i.e. loss of ovarian function before 40 years of age) to 266 premenopausal women, all aged > 45 years, and found an association of POI with higher adiposity and higher CRP levels [40]. Similarly, Honigberg et al. in a study with 144260 postmenopausal women (natural or surgical menopause) found that premature menopause was associated with a small but increased risk for a composite of different CVD [38]. Our study, whilst analysing different parameters, has some consistency with those findings in suggesting that reproductive age associates with intermediate risk factors of CVD, such as adiposity and higher CRP and glucose levels, which could be relevant for later CVD.

Our findings are broadly in line with the narrative review behind the recently published American Heart Association (AHA) statement on menopausal transition and CVD [41]. In that review consistent with our findings, they do not find strong evidence that menopausal transition influences blood pressure or CIMT beyond chronological age and that there is evidence of an increase in fat mass through the menopausal transition, independent of chronological age, as well as fasting glucose, as we also find. They note that non-HDL-c increases across the menopause transition, which we also observed. Notably, they do not discuss in detail magnitudes of change and our review of key papers for this study suggest that these are modest (as in our study). They conclude that guidelines for CVD prevention should have specific reference to the menopause. They highlight the importance of early age at menopause as a risk factor for CVD and that those with surgical menopause, early menopause, and vasomotor symptoms should be considered for exogenous hormone replacement therapy. Previous cohort studies show that premature menopause is associated with CVD after adjustment for age and other CVD risk factors such as high blood pressure [38, 39]. The main aim of our paper adds to this work by using detailed repeat measures of established risk traits to show how these vary in relation to chronological and reproductive age. Whilst we show that chronological age seems to be more important for some risk factors, it is possible that the impact of reproductive age is influenced by those with premature menopause or early menopause. The previous studies were very large (*N* = 144,000 and 301,000) to have power to compare risk of different cardiovascular diseases between premature menopause and menopause aged 50–51 [39] or postmenopausal women without premature menopause [38]. Though the cited studies have much bigger sample sizes, we have repeat data and are able to separate the influence of both chronological and reproductive age. Furthermore, we did not find any evidence of non-linearity between reproductive or chronological age and many outcomes, suggesting that those with an earlier menopause did not appear to over influence our results. We do however note that it may not have been possible to pick this up in our sample. Furthermore, previous studies have found that changes in CVD risk factors over time were similar in women with natural and surgical menopause [34, 35], which supports our findings that chronological age might influence CVD risk more than reproductive age. In relation to the menopausal transition, they note that firm conclusions are difficult to make on the basis of current evidence but suggest supporting women to make behavioural changes (e.g. diet and physical activity) to maintain a healthy weight across mid-life would be potentially beneficial.

## Conclusions

Our study adds importantly to the limited research in this area and, taken together with the small number of previous, generally smaller studies, suggests that reproductive age, defined as time since FMP (independent of chronological age), is unlikely to have a major impact on sub-clinical atherosclerosis but may increase adiposity and risk of type 2 diabetes modestly. Women can be reassured that transitioning through the menopause does not appear to increase atherosclerosis, contrary to widely held beliefs by many health care professionals and the media. The increase in fat mass and fasting glucose with advancing reproductive and chronological age, as well as increase in CIMT and its risk factors with chronological age in midlife, highlights the importance of cardiovascular monitoring in women and support to maintain healthy behaviours.

## Supplementary Information

**Additional file 1: Table S1.** Characteristics of model fit. **Table S2.** Characteristics of imputed data. **Table S3.** Number of participants at each visit. **Table S4.** Characteristics by menopausal stage at each clinic.

**Additional file 2: Table S5.** Main analysis: unadjusted and adjusted models. **Table S6.** Mean difference in outcome by menopausal status. **Table S7.** Predicted mean differences at ages 45, 50 and 55 by menopausal status. **Table S8.** Mediation analysis. **Figure S1.** Shape of non-linear trajectories: fat mass and non-HDL-c. **Figure S2.** Main analysis: unadjusted and adjusted models. **Figure S3.** Sensitivity analyses: main model (multilevel) compared to general estimating equation model and sensitivity to number of visits. **Figure S4.** Sensitivity analyses: excluding women with a plaque.

**Additional file 3: Table S9.** Comparison of age and cardiometabolic risk factors between women included/excluded. **Table S10.** Comparison of confounders between women included/excluded.

**Additional file 4: Table S11.** Summary of relevant longitudinal studies.

## Data Availability

The data that support the findings of this study are available from the ALSPAC executives, but restrictions apply to the availability of these data, which were used under licence for the current study, and so are not publicly available. The ALSPAC study website (http://www.bristol.ac.uk/alspac/researchers/our-data/) contains details of all the data that are available through a fully searchable data dictionary and variable search tool. The analysis plan can be found on the github: https://github.com/gc13313/Menopause-and-CV-health. The European Union’s Horizon 2020 research and innovation programme under grant agreement No 874739 (LongITools) funds AGS' salary.

## Abbreviations

AHA: American Heart Association
AIC: Akaike Information Criterion
ALSPAC: Avon Longitudinal Study of Parents and Children
BIC: Bayesian Information Criterion
BMI: Body mass index
CIMT: Carotid-intima media thickness
CRP: C-reactive protein
CSE: Certificate of Secondary Education
CVD: Cardiovascular disease
DBP: Diastolic blood pressure
FMP: Final menstrual period
GEE: General estimating equations
HDL-c: High-density lipoprotein cholesterol
HRT: Hormone replacement therapy
IQR: Interquartile range
MAR: Missing at random
POI: Premature ovarian insufficiency
RCT: Randomised controlled trial
SBP: Systolic blood pressure
STRAW: Stages of Reproductive Aging Workshop
