# Supplementary material for: Cardiovascular health in the menopause transition: a longitudinal study of up to 3892 women with up to four repeated measures of risk factors
Source: BMC Med. 2022 Aug 17;20:299. doi: 10.1186/s12916-022-02454-6 (PMC9382827; doi:10.1186/s12916-022-02454-6)
Supplement: Supplementary file 1 — Additional file 1: Table S1. Characteristics of model fit. Table S2. Characteristics of imputed data. Table S3. Number of participants at each visit. Table S4. Characteristics by menopausal stage at each clinic. [file 12916_2022_2454_MOESM1_ESM.docx]

# Additional file 1

# Contents

- Table S1 Characteristics of the model fit for each cardiovascular measure according to reproductive and chronological age in analysis population 1 (N= 1702 women)
- Table S2 Characteristics of the imputed data before and after imputation
- Table S3 Number of participants with data on each outcome at each clinic visit and overall
- Table S4 Distributions of chronological age and all outcomes by menopausal stage at each clinic (N= 3892 women)

# Supplementary Tables and Figures

**Table S1** Characteristics of the model fit for each cardiovascular measure according to reproductive and chronological age in analysis population 1 (N= 1702 women)

| Outcome | Model | N participants | N Observations | DF | Deviance | AIC | BIC |
| --- | --- | --- | --- | --- | --- | --- | --- |
| BMI | Reproductive age unadjusted | 1699 | 4720 | 6 | -10577 | 21166 | 21205 |
|  | Age unadjusted |  |  | 6 | -10600 | 21212 | 21251 |
|  | Mutually adjusted |  |  | 7 | -10598 | 21211 | 21256 |
| Lean mass | Reproductive age unadjusted | 1682 | 4622 | 6 | -6089 | 12189 | 12228 |
|  | Age unadjusted |  |  | 6 | -6089 | 12191 | 12229 |
|  | Mutually adjusted |  |  | 7 | -6089 | 12191 | 12236 |
| Fat mass | Reproductive age unadjusted | 1682 | 4622 | 6 | -9646 | 19304 | 19343 |
|  | Age unadjusted |  |  | 6 | -9672 | 19357 | 19395 |
|  | Mutually adjusted |  |  | 7 | -9665 | 19344 | 19390 |
| SBP | Reproductive age unadjusted | 1692 | 4700 | 5 | -18032 | 36074 | 36106 |
|  | Age unadjusted |  |  | 5 | -18012 | 36035 | 36067 |
|  | Mutually adjusted |  |  | 6 | -18012 | 36036 | 36075 |
| DBP | Reproductive age unadjusted | 1692 | 4700 | 5 | -15968 | 31946 | 31978 |
|  | Age unadjusted |  |  | 5 | -15963 | 31936 | 31969 |
|  | Mutually adjusted |  |  | 6 | -15962 | 31937 | 31976 |
| Pulse rate | Reproductive age unadjusted | 1692 | 4700 | 5 | -16385 | 32779 | 32811 |
|  | Age unadjusted |  |  | 5 | -16387 | 32784 | 32817 |
|  | Mutually adjusted |  |  | 6 | -16375 | 32763 | 32802 |
| Triglycerides | Reproductive age unadjusted | 1638 | 4456 | 6 | -1172 | 2356 | 2394 |
|  | Age unadjusted |  |  | 6 | -1165 | 2342 | 2381 |
|  | Mutually adjusted |  |  | 7 | -1164 | 2343 | 2388 |
| Non-HDL-c | Reproductive age unadjusted | 1638 | 4456 | 7 | -4345 | 8704 | 8749 |
|  | Age unadjusted |  |  | 6 | -4321 | 8654 | 8693 |
|  | Mutually adjusted |  |  | 8 | -4303 | 8622 | 8674 |
| HDL-c | Reproductive age unadjusted | 1638 | 4456 | 6 | -86 | 185 | 223 |
|  | Age unadjusted |  |  | 6 | -84 | 179 | 217 |
|  | Mutually adjusted |  |  | 7 | -76 | 166 | 211 |
| CRP | Reproductive age unadjusted | 1628 | 4276 | 6 | -5333 | 10678 | 10716 |
|  | Age unadjusted |  |  | 6 | -5340 | 10691 | 10729 |
|  | Mutually adjusted |  |  | 7 | -5333 | 10680 | 10724 |
| Glucose | Reproductive age unadjusted | 1638 | 4454 | 6 | 3158 | -6304 | -6266 |
|  | Age unadjusted |  |  | 6 | 3151 | -6290 | -6252 |
|  | Mutually adjusted |  |  | 7 | 3154 | -6293 | -6248 |

BMI: body mass index; SBP: systolic blood pressure; DBP: diastolic blood pressure; Non-HDL-c: Non-high-density lipoprotein-cholesterol; HDL-c: high-density lipoprotein-cholesterol; CRP: C-reactive protein.

**Table S2** Characteristics of the imputed data before and after imputation*

|  | % missing | Before imputation  % | After imputation  % |
| --- | --- | --- | --- |
| Pre/early pregnancy BMI | 12.1 | 1496 |  |
| Normal |  | 86.0 | 85.5 |
| Overweight |  | 11.4 | 11.8 |
| Obese |  | 2.6 | 2.7 |
| Smoking status | 12.8 | 1483 |  |
| Never smoker |  | 54.6 | 53.3 |
| Former smoker |  | 35.4 | 35.8 |
| Current smoker |  | 10.0 | 10.8 |
| Alcohol intake frequency | 32.1 | 1155 |  |
| Never or less than 4x/ month |  | 40.5 | 40.8 |
| 2 to 3 times a week |  | 33.2 | 32.6 |
| 4 or more times a week |  | 26.2 | 26.6 |
| Age at menarche | 11.4 | 1507 |  |
| Early (≤ 11 years) |  | 16.1 | 17.1 |
| Normal (12-14 years) |  | 70.0 | 69.2 |
| Late (≥ 15 years) |  | 13.9 | 13.7 |
| Educational achievement | 6.7 | 1587 |  |
| CSE / Vocational degree/ O-level |  | 43.4 | 43.8 |
| A-level |  | 31.4 | 31.2 |
| University degree |  | 25.3 | 25.0 |

* All other outcomes had <5% missing data. A-level: advanced level; BMI: body mass index; CSE: certificate of secondary education; O-level: ordinary level.

**Table S3** Number of participants with data on each outcome at each clinic visit and overall

|  |  | |  | |  | |  |  | Total N/Observations | | | |  | |
| --- | --- | --- | --- | --- | --- | --- | --- | --- | --- | --- | --- | --- | --- | --- |
| Outcomes | Clinic 1 | | Clinic 2 | | Clinic 3 | | Clinic 4 |  | Before imputation | | After imputation | | Percentage missing | |
| BMI | 1520/1524 | 99.7% | 1077/1082 | 99.5% | 1088/1091 | 99.7% | 1036/1037 | 99.9% | 1699 | 4720 | 1702 | 4734 | | 0.2 |
| Lean mass | 1501/1524 | 98.5% | 1045/1082 | 96.6% | 1064/1091 | 97.5% | 1014/1037 | 97.8% | 1682 | 4622 | 1702 | 4734 | | 1.2 |
| Fat mass | 1501/1524 | 98.5% | 1045/1082 | 96.6% | 1064/1091 | 97.5% | 1014/1037 | 97.8% | 1682 | 4622 | 1702 | 4734 | | 1.2 |
| SBP | 1509/1524 | 99.0% | 1075/1082 | 99.4% | 1084/1091 | 99.4% | 1032/1037 | 99.5% | 1692 | 4700 | 1702 | 4734 | | 0.6 |
| DBP | 1509/1524 | 99.0% | 1075/1082 | 99.4% | 1084/1091 | 99.4% | 1032/1037 | 99.5% | 1692 | 4700 | 1702 | 4734 | | 0.6 |
| Pulse rate | 1509/1524 | 99.0% | 1075/1082 | 99.4% | 1084/1091 | 99.4% | 1032/1037 | 99.5% | 1692 | 4700 | 1702 | 4734 | | 0.6 |
| Triglycerides | 1407/1524 | 92.3% | 1030/1082 | 95.2% | 1041/1091 | 95.4% | 978/1037 | 94.3% | 1638 | 4456 | 1702 | 4734 | | 3.8 |
| HDL-c | 1407/1524 | 92.3% | 1030/1082 | 95.2% | 1041/1091 | 95.4% | 978/1037 | 94.3% | 1638 | 4456 | 1702 | 4734 | | 3.8 |
| Non-HD-c | 1407/1524 | 92.3% | 1030/1082 | 95.2% | 1041/1091 | 95.4% | 978/1037 | 94.3% | 1638 | 4456 | 1702 | 4734 | | 3.8 |
| CRP | 1407/1524 | 92.3% | 1028/1082 | 95.0% | 1041/1091 | 95.4% | 978/1037 | 94.3% | 1628 | 4276 | 1702 | 4734 | | 4.3 |
| Glucose | 1407/1524 | 92.3% | 964/1082 | 89.1% | 991/1091 | 90.8% | 914/1037 | 88.1% | 1638 | 4454 | 1702 | 4734 | | 3.8 |

BMI: body mass index; SBP: systolic blood pressure; DBP: diastolic blood pressure; Non-HDL-c: Non-high-density lipoprotein-cholesterol; HDL-c: high-density lipoprotein-cholesterol; CRP: C-reactive protein.

# Table S4 Distributions of chronological age and all outcomes by menopausal stage at each clinic (N= 3892 women)

|  | 1^st^ assessment | 2^nd^ assessment | 3^rd^ assessment | 4^th^ assessment |
| --- | --- | --- | --- | --- |
| Menopausal stage N(%) |  |  |  |  |
| Premenopause | 2198/3560 (61.7%) | 825/2070 (39.9%) | 641/2143 (29.9%) | 454/2068 (22.0%) |
| Perimenopause | 701/3560 (19.7%) | 551/2070 (26.6%) | 583/2143 (27.2%) | 553/2068 (26.7%) |
| Postmenopause | 661/3560 (18.6%) | 694/2070 (33.5%) | 919/2143 (42.9%) | 1061/2068 (51.3%) |
| Age (years): mean (SD) |  |  |  |  |
| All | 47.5 (4.5) | 50.5 (4.4) | 51.8 (4.5) | 52.8 (4.4) |
| Premenopause | 45.2 (3.3) | 47.2 (3.2) | 47.7 (3.2) | 48.2 (3.2) |
| Perimenopause | 49.5 (2.6) | 50.5 (2.7) | 50.9 (2.6) | 51.2 (2.3) |
| Post menopause | 53.1 (3.4) | 54.4 (3.5) | 55.1 (3.5) | 55.6 (3.5) |
| CIMT (mm): median (IQR) |  |  |  |  |
| All | 0.56 (0.52, 0.59) |  |  | 0.60 (0.55, 0.67) |
| Premenopause | 0.55 (0.51, 0.58) |  |  | 0.57 (0.52, 0.65) |
| Perimenopause | 0.57 (0.53, 0.60) |  |  | 0.60 (0.54, 0.66) |
| Postmenopause | 0.57 (0.54, 0.61) |  |  | 0.62 (0.56, 0.69) |
| BMI (kg/m^2^): mean (SD) |  |  |  |  |
| All | 26.4 (5.2) | 26.2 (5.1) | 26.3 (5.1) | 26.2 (5.3) |
| Premenopause | 26.6 (5.3) | 26.5 (5.3) | 26.7 (5.2) | 26.7 (5.4) |
| Perimenopause | 26.0 (5.0) | 26.3 (5.3) | 26.5 (5.3) | 26.7 (5.7) |
| Postmenopause | 26.2 (5.2) | 25.6 (4.6) | 25.8 (4.9) | 25.8 (4.9) |
| Total lean mass (kg/m^2^): mean (SD) | |  |  |  |
| All | 15.3 (1.6) | 15.5 (1.6) | 15.2 (1.6) | 15.2 (1.7) |
| Premenopause | 15.4 (1.6) | 15.7 (1.7) | 15.5 (1.6) | 15.6 (1.6) |
| Perimenopause | 15.2 (1.5) | 15.5 (1.6) | 15.3 (1.6) | 15.4 (1.8) |
| Postmenopause | 15.1 (1.5) | 15.2 (1.5) | 15.0 (1.5) | 14.9 (1.6) |
| Total fat mass (kg/m^2^): mean SD) |  |  |  |  |
| All | 9.9 (4.0) | 9.8 (3.7) | 9.9 (4.0) | 10.2 (3.9) |
| Premenopause | 10.0 (4.0) | 9.8 (3.8) | 10.0 (4.0) | 10.3 (4.1) |
| Perimenopause | 9.7 (3.9) | 9.9 (3.9) | 10.0 (4.1) | 10.4 (4.1) |
| Postmenopause | 10.0 (3.8) | 9.6 (3.3) | 9.6 (3.8) | 10.1 (3.7) |
| SBP (mmHg): mean (SD) |  |  |  |  |
| All | 117.9 (12.4) | 121.3 (14.3) | 118.8 (14.0) | 119.0 (14.4) |
| Premenopause | 117.3 (11.8) | 120.8 (13.7) | 118.1 (13.5) | 117.3 (13.1) |
| Perimenopause | 117.9 (12.8) | 122.5 (15.0) | 119.4 (14.6) | 119.2 (15.0) |
| Postmenopause | 119.6 (13.4) | 121.0 (14.2) | 118.9 (13.9) | 119.7 (14.6) |
| DBP (mmHg): mean (SD) |  |  |  |  |
| All | 71.5 (8.2) | 71.7 (9.6) | 70.2 (9.1) | 70.2 (9.4) |
| Premenopause | 71.2 (8.1) | 71.7 (9.5) | 70.4 (9.4) | 70.3 (9.7) |
| Perimenopause | 71.7 (8.2) | 72.3 (10.0) | 70.5 (9.3) | 70.5 (9.5) |
| Postmenopause | 72.1 (8.3) | 71.1 (9.4) | 69.9 (8.8) | 70.0 (9.2) |
| Pulse rate (bpm): mean (SD) |  |  |  |  |
| All | 67.1 (8.7) | 69.6 (9.6) | 71.2 (10.1) | 69.0 (9.9) |
| Premenopause | 67.4 (8.7) | 70.2 (9.7) | 72.4 (10.2) | 69.5 (10.3) |
| Perimenopause | 66.6 (8.8) | 69.4 (9.5) | 70.4 (10.2) | 69.2 (9.6) |
| Postmenopause | 66.5 (8.5) | 69.1 (9.5) | 71.0 (9.8) | 68.6 (9.9) |
| Non-HDL-c (mmol/l): mean (SD) |  |  |  |  |
| All | 3.4 (0.9) | 3.5 (0.8) | 3.6 (0.8) | 3.8 (0.9) |
| Premenopause | 3.3 (0.8) | 3.4 (0.7) | 3.4 (0.8) | 3.5 (0.8) |
| Perimenopause | 3.5 (0.9) | 3.5 (0.8) | 3.5 (0.8) | 3.8 (0.8) |
| Postmenopause | 3.8 (1.0) | 3.7 (0.9) | 3.8 (0.9) | 4.0 (0.9) |
| HDL-c: mean (SD) |  |  |  |  |
| All | 1.5 (0.4) | 1.6 (0.3) | 1.6 (0.3) | 1.6 (0.4) |
| Premenopause | 1.5 (0.4) | 1.5 (0.3) | 1.5 (0.3) | 1.5 (0.3) |
| Perimenopause | 1.6 (0.4) | 1.6 (0.3) | 1.6 (0.3) | 1.6 (0.3) |
| Postmenopause | 1.6 (0.4) | 1.7 (0.3) | 1.6 (0.4) | 1.6 (0.4) |
| Triglycerides (mmol/l): median (IQR) | |  |  |  |
| All | 0.9 (0.7, 1.2) | 0.9 (0.7, 1.2) | 0.8 (0.7, 1.1) | 0.9 (0.7, 1.2) |
| Premenopause | 0.8 (0.7, 1.1) | 0.8 (0.7, 1.1) | 0.8 (0.6, 1.1) | 0.9 (0.7, 1.2) |
| Perimenopause | 0.9 (0.7, 1.2) | 0.9 (0.7, 1.2) | 0.8 (0.7, 1.1) | 0.9 (0.7, 1.2) |
| Postmenopause | 0.9 (0.7, 1.3) | 0.9 (0.7, 1.3) | 0.8 (0.7, 1.2) | 0.9 (0.7, 1.3) |
| Glucose (mmol/l): median (IQR) |  |  |  |  |
| All | 5.2 (4.9, 5.5) | 5.2 (5.0, 5.5) | 5.1 (4.9, 5.4) | 5.3 (5.0, 5.6) |
| Premenopause | 5.2 (4.9, 5.4) | 5.2 (4.9, 5.4) | 5.1 (4.9, 5.4) | 5.2 (5.0, 5.5) |
| Perimenopause | 5.1 (4.9, 5.4) | 5.2 (4.9, 5.4) | 5.1 (4.9, 5.4) | 5.3 (5.0, 5.5) |
| Postmenopause | 5.2 (5.0, 5.5) | 5.3 (5.0, 5.5) | 5.2 (4.9, 5.5) | 5.3 (5.1, 5.6) |
| CRP (mg/l),: median (IQR) |  |  |  |  |
| All | 0.9 (0.4, 2.0) | 1.0 (0.5, 2.2) | 1.0 (0.5, 2.3) | 1.1 (0.5, 2.4) |
| Premenopause | 0.9 (0.4, 2.0) | 1.0 (0.5, 2.2) | 0.9 (0.5, 2.3) | 1.1 (0.5, 2.5) |
| Perimenopause | 0.9 (0.4, 1.9) | 1.0 (0.5, 2.4) | 1.0 (0.6, 2.3) | 1.1 (0.6, 2.3) |
| Postmenopause | 1.1 (0.5, 2.2) | 1.0 (0.5, 2.1) | 1.0 (0.5, 2.2) | 1.1 (0.5, 2.4) |

BMI: body mass index; SBP: systolic blood pressure; DBP: diastolic blood pressure; Non-HDL-c: Non-high-density lipoprotein-cholesterol; HDL-c: high-density lipoprotein-cholesterol; CRP: C-reactive protein.
