# Supplementary material for: Cardiovascular health in the menopause transition: a longitudinal study of up to 3892 women with up to four repeated measures of risk factors
Source: BMC Med. 2022 Aug 17;20:299. doi: 10.1186/s12916-022-02454-6 (PMC9382827; doi:10.1186/s12916-022-02454-6)

# Additional file 2

# Contents

- Table S5: Associations of reproductive and chronological aging with all cardiometabolic measures: unadjusted and adjusted models
- Figure S1 Average predicted mean (95% CI) for fat mass (reproductive age to the power of 0.5) and non-HDL-c (linear and cubic term for reproductive age) across reproductive age (for a woman aged 50 years, at mean pre-pregnancy BMI and the reference categories of all categorical confounders)
- Figure S2 Associations of reproductive and chronological aging with all cardiometabolic measures: unadjusted and adjusted models
- Table S6 Mean difference in outcome (per year in chronological age) by menopausal status (pre, peri or post)
- Table S7 Predicted mean differences at (chronological) ages 45, 50 and 55 by menopausal status
- Table S8 Mediation analysis of the association between chronological age and CIMT by changes in risk factors
- Figure S3 Associations of reproductive and chronological aging with all cardiometabolic measures: sensitivity analyses
- Figure S4 Associations of reproductive and chronological aging with all cardiometabolic measures: sensitivity analysis excluding women with a plaque

# Table S5 Associations of reproductive and chronological aging with all cardiometabolic measures: unadjusted and adjusted models

|  | Reproductive age  MD [95% CI]  p-value | | | Chronological age  MD [95% CI]  p-value | | |
| --- | --- | --- | --- | --- | --- | --- |
|  | Unadjusted | Adjusted for chronological age | Adjusted for measured confounders* | Unadjusted | Adjusted for reproductive age | Adjusted for measured confounders* |
| CIMT (μm) | 7.58 [6.52,8.64] | 1.01 [-0.23,2.25] | 0.84 [-0.42,2.09] | 8.01 [7.17,8.86] | 7.30 [6.07,8.53] | 7.58 [6.31,8.86] |
|  | <0.001 | 0.1099 | 0.1931 | <0.001 | <0.001 | <0.001 |
| BMI (kg/m^2^) | -0.00 [-0.02,0.02] | 0.06 [0.01,0.12] | 0.03 [-0.01,0.07] | -0.01 [-0.03,0.01] | -0.07 [-0.13,-0.01] | -0.04 [-0.08,0.01] |
|  | 0.8780 | 0.0295 | 0.2022 | 0.4478 | 0.0214 | 0.1077 |
| Total lean mass (kg/m^2^) | -0.03 [-0.04,-0.02] | -0.01 [-0.03,0.01] | -0.02 [-0.03,-0.00] | -0.029 [-0.037,-0.021] | -0.017 [-0.035,0.001] | -0.016 [-0.031,-0.001] |
|  | <0.001 | 0.1573 | 0.0263 | <0.001 | 0.0614 | 0.0387 |
| Total fat mass (kg/m^2^) | 0.07 [0.05,0.09] | 0.10 [0.06,0.15] | 0.06 [0.03,0.10] | 0.06 [0.04,0.08] | -0.02 [-0.06,0.02] | -0.00 [-0.03,0.03] |
|  | <0.001 | <0.001 | 0.0004 | <0.001 | 0.3095 | 0.8576 |
| SBP (mmHg) | 0.21 [0.10,0.31] | -0.04 [-0.19,0.12] | -0.10 [-0.26,0.06] | 0.27 [0.18,0.37] | 0.30 [0.15,0.45] | 0.37 [0.21,0.53] |
|  | 0.0001 | 0.6190 | 0.2105 | <0.001 | 0.0001 | 0.0000 |
| DBP (mmHg) | -0.18 [-0.25,-0.12] | -0.08 [-0.18,0.02] | -0.11 [-0.21,-0.01] | -0.18 [-0.24,-0.12] | -0.12 [-0.22,-0.02] | -0.08 [-0.19,0.02] |
|  | <0.001 | 0.1155 | 0.0370 | 0.0000 | 0.0162 | 0.1099 |
| Pulse rate (bpm) | 0.39 [0.32,0.46] | 0.32 [0.21,0.42] | 0.24 [0.12,0.35] | 0.35 [0.28,0.41] | 0.10 [-0.01,0.20] | 0.19 [0.08,0.30] |
|  | <0.001 | <0.001 | <0.001 | <0.001 | 0.0640 | 0.0011 |
| Triglycerides (mmol/l), logged | 0.01 [0.01,0.02] | 0.00 [-0.00,0.01] | 0.00 [-0.00,0.01] | 0.013 [0.010,0.016] | 0.010 [0.005,0.015] | 0.013 [0.008,0.018] |
|  | <0.001 | 0.1717 | 0.8984 | <0.001 | 0.0001 | 0.0000 |
| Non-HDL-c (mmol/l) | 0.07 [0.06,0.07] | 0.02 [0.01,0.03] | 0.02 [0.005,0.03] | 0.07 [0.06,0.08] | 0.05 [0.04,0.06] | 0.05 [0.04,0.07] |
|  | <0.001 | 0.0003 | 0.0059 | <0.001 | <0.001 | <0.001 |
| HDL-c (mmol/l) | 0.011 [0.009,0.014] | 0.001 [-0.004,0.005] | 0.003 [-0.001,0.007] | 0.012 [0.010,0.015] | 0.007 [0.003,0.011] | 0.005 [0.001,0.009] |
|  | <0.001 | 0.7820 | 0.1810 | <0.001 | 0.0013 | 0.0076 |
| CRP (mg/l), logged | 0.02 [0.01,0.03] | 0.02 [0.01,0.04] | 0.01 [0.00,0.02] | 0.02 [0.01,0.03] | 0.00 [-0.01,0.01] | 0.01 [-0.00,0.02] |
|  | <0.001 | 0.0005 | 0.0282 | <0.001 | 0.9335 | 0.1158 |
| Glucose (mmol/l), logged | 0.003 [0.002,0.004] | 0.002 [0.000,0.003] | 0.002 [0.000,0.003] | 0.003 [0.002,0.004] | 0.001 [-0.000,0.003] | 0.002 [0.000,0.003] |
|  | <0.001 | 0.0178 | 0.0488 | <0.001 | 0.0602 | 0.0379 |

* mutual adjustment for each age variable and pre-pregnancy BMI, age at menarche, parity, maternal education, smoking status and alcohol intake

**Figure S1** Average predicted mean (95% CI) for fat mass (reproductive age to the power of 0.5) and non-HDL-c (linear and cubic term for reproductive age) across reproductive age (for a woman aged 50 years, at mean pre-pregnancy BMI and the reference categories of all categorical confounders)

# Figure S2 Associations of reproductive and chronological aging with all cardiometabolic measures: unadjusted and adjusted models


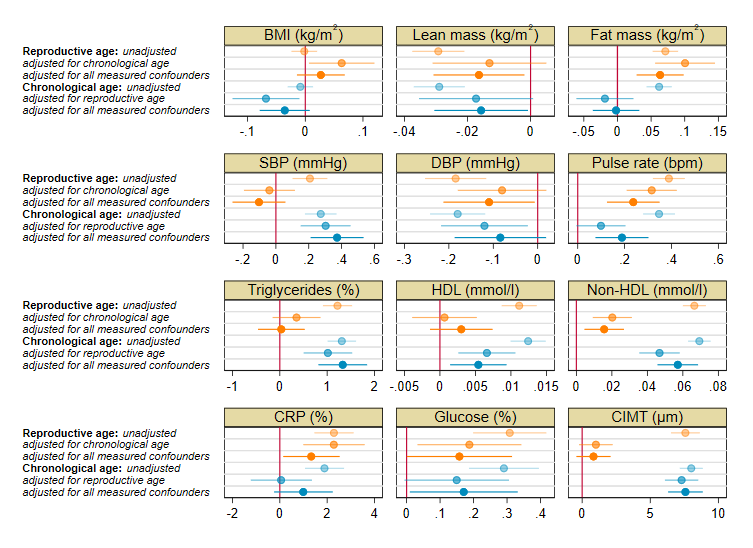


# Table S6 Mean difference in outcome (per year in chronological age) by menopausal status (pre, peri or post) (N=3892)

| Outcome/ CVD risk factor | Pre-menopause | p-value | Peri-menopause | p-value | Post-menopause | p-value | p-value for interaction between age and menopausal status |
| --- | --- | --- | --- | --- | --- | --- | --- |
| CIMT (μm) | 6.12 [5.04,7.20] | <0.001 | 8.47 [6.47,10.46] | <0.001 | 8.13 [6.89,9.38] | <0.001 | 0.019 |
| BMI (kg/m^2^) | 0.00  [-0.02,0.03] | 0.909 | -0.01  [-0.04,0.02] | 0.615 | -0.04  [-0.07,-0.01] | 0.004 | 0.048 |
| Total lean mass (kg/m^2^) | 0.01 [0.00,0.02] | 0.003 | -0.01  [-0.02,0.00] | 0.055 | -0.03  [-0.04,-0.02] | <0.001 | <0.001 |
| Total fat mass (kg/m^2^) | 0.01  [-0.01,0.03] | 0.494 | 0.04 [0.01,0.07] | 0.003 | 0.02  [-0.00,0.04] | 0.080 | 0.066 |
| SBP (mmHg) | 0.40 [0.28,0.51] | <0.001 | 0.22 [0.06,0.38] | 0.006 | 0.23 [0.10,0.36] | <0.001 | 0.068 |
| DBP (mmHg) | 0.03  [-0.05,0.10] | 0.478 | -0.07  [-0.18,0.04] | 0.214 | -0.21  [-0.30,-0.13] | <0.001 | <0.001 |
| Pulse rate (bpm) | 0.31 [0.22,0.39] | <0.001 | 0.21 [0.09,0.32] | <0.001 | 0.28 [0.19,0.37] | <0.001 | 0.316 |
| Triglycerides (%) | 0.65 [0.30,1.00] | <0.001 | 1.02 [0.52,1.52] | <0.001 | 0.94 [0.56,1.32] | <0.001 | 0.346 |
| HDL-c (mmol/l) | 0.02 [0.01,0.02] | <0.001 | 0.01 [0.01,0.02] | <0.001 | 0.01 [0.01,0.01] | <0.001 | 0.071 |
| Non-HDL-c (mmol/l) | 0.03 [0.03,0.04] | <0.001 | 0.06 [0.05,0.07] | <0.001 | 0.05 [0.05,0.06] | <0.001 | <0.001 |
| CRP (%) | 1.36 [0.47,2.27] | 0.003 | 1.44 [0.10,2.79] | 0.034 | 1.23 [0.23,2.24] | 0.016 | 0.963 |
| Glucose (%) | 0.11  [-0.00,0.23] | 0.055 | 0.29 [0.12,0.46] | 0.001 | 0.26 [0.14,0.39] | <0.001 | 0.100 |

Associations interpreted as mean difference in outcome per year in chronological age in each menopausal stage (pre-, peri- or post-menopause).

# Table S7 Predicted mean differences at (chronological) ages 45, 50 and 55 by menopausal status

|  | Age | Pre | Peri | Post | Peri vs pre | p-value | Post vs pre | p-value |
| --- | --- | --- | --- | --- | --- | --- | --- | --- |
| CIMT (μm) | 45 | 554.4 (543.0,565.8) | 552.0 (536.2,567.9) | 549.4 (532.8,565.9) | -2.3 (-14.5,9.8) | 0.706 | -5.0 (-18.1,8.1) | 0.453 |
|  | 50 | 584.9 (572.3,597.6) | 594.3 (582.2,606.5) | 590.0 (576.9,603.1) | 9.4 (1.8,17.0) | 0.016 | 5.1 (-4.2,14.3) | 0.283 |
|  | 55 | 615.5 (599.7,631.3) | 636.7 (621.1,652.3) | 630.7 (618.7,642.7) | 21.1 (6.6,35.7) | 0.004 | 15.1 (3.8,26.5) | 0.009 |
| BMI (kg/m^2^) | 45 | 27.3 (26.8,27.8) | 27.5 (27.0,28.0) | 27.7 (27.2,28.3) | 0.4 (0.2,0.7) | 0.001 | 0.2 (-0.0,0.4) | 0.096 |
|  | 50 | 27.3 (26.8,27.8) | 27.4 (27.0,27.9) | 27.6 (27.1,28.0) | 0.2 (0.1,0.4) | 0.008 | 0.1 (0.0,0.2) | 0.050 |
|  | 55 | 27.3 (26.8,27.9) | 27.4 (26.9,27.9) | 27.4 (26.9,27.8) | 0.0 (-0.2,0.2) | 0.833 | 0.1 (-0.1,0.3) | 0.530 |
| Lean mass (kg/m^2^) | 45 | 15.5 (15.3,15.6) | 15.5 (15.3,15.7) | 15.4 (15.3,15.6) | -0.0 (-0.1,0.1) | 0.486 | 0.0 (-0.1,0.1) | 0.670 |
|  | 50 | 15.5 (15.4,15.7) | 15.4 (15.3,15.6) | 15.3 (15.1,15.5) | -0.2 (-0.3,-0.2) | 0.000 | -0.1 (-0.2,-0.1) | 0.000 |
|  | 55 | 15.6 (15.4,15.8) | 15.4 (15.2,15.5) | 15.2 (15.0,15.3) | -0.4 (-0.5,-0.3) | 0.000 | -0.2 (-0.3,-0.1) | 0.000 |
| Fat mass (kg/m^2^) | 45 | 10.8 (10.4,11.2) | 10.8 (10.4,11.2) | 11.1 (10.6,11.5) | 0.0 (-0.1,0.2) | 0.773 | 0.3 (0.1,0.5) | 0.006 |
|  | 50 | 10.8 (10.4,11.2) | 11.0 (10.6,11.4) | 11.2 (10.8,11.6) | 0.2 (0.1,0.3) | 0.000 | 0.4 (0.2,0.5) | 0.000 |
|  | 55 | 10.8 (10.4,11.3) | 11.2 (10.8,11.6) | 11.3 (10.9,11.7) | 0.4 (0.2,0.5) | 0.000 | 0.4 (0.3,0.6) | 0.000 |
| SBP (mmHg) | 45 | 118.3 (116.7,119.9) | 119.0 (117.2,120.8) | 118.6 (116.7,120.6) | 0.7 (-0.3,1.7) | 0.194 | 0.3 (-1.0,1.6) | 0.616 |
|  | 50 | 120.3 (118.6,121.9) | 120.1 (118.5,121.7) | 119.8 (118.1,121.5) | -0.2 (-0.8,0.4) | 0.516 | -0.5 (-1.4,0.3) | 0.242 |
|  | 55 | 122.3 (120.4,124.2) | 121.2 (119.4,123.0) | 120.9 (119.3,122.6) | -1.1 (-2.2,0.1) | 0.065 | -1.3 (-2.4,-0.3) | 0.015 |
| DBP (mmHg) | 45 | 71.7 (70.6,72.7) | 72.2 (71.0,73.4) | 73.2 (71.9,74.5) | 0.5 (-0.1,1.2) | 0.118 | 1.5 (0.7,2.4) | 0.000 |
|  | 50 | 71.8 (70.7,72.9) | 71.9 (70.8,72.9) | 72.1 (71.0,73.2) | 0.1 (-0.4,0.5) | 0.781 | 0.3 (-0.2,0.9) | 0.263 |
|  | 55 | 71.9 (70.7,73.2) | 71.5 (70.3,72.7) | 71.1 (70.0,72.1) | -0.4 (-1.2,0.4) | 0.288 | -0.9 (-1.6,-0.1) | 0.019 |
| Pulse rate (bpm) | 45 | 68.8 (67.6,69.9) | 68.8 (67.5,70.1) | 67.9 (66.5,69.4) | 0.0 (-0.7,0.8) | 0.917 | -0.8 (-1.7,0.1) | 0.089 |
|  | 50 | 70.3 (69.1,71.5) | 69.8 (68.6,71.0) | 69.4 (68.1,70.6) | -0.5 (-0.9,-0.0) | 0.049 | -0.9 (-1.6,-0.3) | 0.003 |
|  | 55 | 71.8 (70.4,73.2) | 70.9 (69.6,72.2) | 70.8 (69.6,72.0) | -0.9 (-1.8,-0.1) | 0.027 | -1.0 (-1.8,-0.3) | 0.009 |
| Triglycerides (mmol/l) | 45 | 0.9 (0.9,1.0) | 0.9 (0.9,1.0) | 1.0 (0.9,1.0) | -0.00 (-0.03,0.03) | 0.942 | 0.02 (-0.02,0.05) | 0.421 |
|  | 50 | 1.0 (0.9,1.0) | 1.0 (0.9,1.0) | 1.0 (0.9,1.1) | 0.02 (-0.00,0.04) | 0.080 | 0.03 (0.00,0.06) | 0.022 |
|  | 55 | 1.0 (0.9,1.1) | 1.0 (1.0,1.1) | 1.0 (1.0,1.1) | 0.04 (-0.00,0.07) | 0.055 | 0.04 (0.01,0.08) | 0.008 |
| HDL (mmol/l) | 45 | 1.4 (1.3,1.4) | 1.4 (1.4,1.5) | 1.4 (1.4,1.5) | 0.05 (0.02,0.08) | 0.000 | 0.04 (0.01,0.06) | 0.001 |
|  | 50 | 1.4 (1.4,1.5) | 1.5 (1.4,1.5) | 1.5 (1.4,1.5) | 0.03 (0.01,0.05) | 0.002 | 0.03 (0.02,0.05) | 0.000 |
|  | 55 | 1.5 (1.5,1.6) | 1.6 (1.5,1.6) | 1.5 (1.5,1.6) | 0.01 (-0.01,0.03) | 0.448 | 0.03 (0.00,0.05) | 0.032 |
| Non-HDL (mmol/l) | 45 | 3.3 (3.2,3.4) | 3.3 (3.2,3.5) | 3.5 (3.3,3.6) | 0.1 (0.1,0.2) | 0.000 | 0.0 (-0.1,0.1) | 0.904 |
|  | 50 | 3.5 (3.4,3.6) | 3.6 (3.5,3.7) | 3.7 (3.6,3.8) | 0.2 (0.2,0.3) | 0.000 | 0.1 (0.1,0.2) | 0.000 |
|  | 55 | 3.7 (3.6,3.8) | 3.9 (3.8,4.0) | 4.0 (3.9,4.1) | 0.3 (0.3,0.4) | 0.000 | 0.2 (0.2,0.3) | 0.000 |
|  | 45 | 1.3 (1.1,1.4) | 1.3 (1.2,1.6) | 1.4 (1.2,1.6) | 0.06 (-0.03,0.14) | 0.186 | 0.08 (-0.02,0.18) | 0.118 |
| CRP (mg/l) | 50 | 1.4 (1.2,1.5) | 1.4 (1.3,1.6) | 1.5 (1.3,1.7) | 0.06 (0.01,0.11) | 0.025 | 0.07 (0.01,0.14) | 0.035 |
|  | 55 | 1.5 (1.3,1.7) | 1.6 (1.3,1.8) | 1.6 (1.4,1.8) | 0.06 (-0.03,0.16) | 0.192 | 0.07 (-0.02,0.15) | 0.130 |
| Glucose (mmol/l) | 45 | 5.2 (5.1,5.3) | 5.2 (5.1,5.3) | 5.2 (5.1,5.3) | -0.01 (-0.02,0.00) | 0.236 | 0.00 (-0.01,0.01) | 0.931 |
|  | 50 | 5.2 (5.2,5.3) | 5.2 (5.2,5.3) | 5.3 (5.2,5.4) | 0.00 (-0.00,0.01) | 0.485 | 0.01 (-0.00,0.02) | 0.075 |
|  | 55 | 5.3 (5.2,5.4) | 5.3 (5.2,5.4) | 5.3 (5.3,5.4) | 0.01 (-0.00,0.02) | 0.081 | 0.02 (0.00,0.03) | 0.006 |

Predicted mean differences in outcome per year in chronological age in the peri-menopause or post-menopause group (compared to the pre-menopause group) to show where the differences between groups occur based on the menopause by age interaction in Figures 2 and 3. Predictions in each group at (chronological) age at 45, 50 and 55 years based on a women with pre-pregnancy BMI of 22.4, one child, never smoker, never or drink less than 4 times per week, maternal education of CSE / Vocational degree/ O-level and had an early age at menarche (<12 years). Triglycerides, CRP and glucose differences presented on the log scale.

For CIMT, analyses were based on the following number of observations in the (pre, peri, post) groups for the ≤40, >40-≤45, >45-≤50, >50-≤55, >55-≤60 and >60 age groups respectively = (188, 4, 1), (1100, 21, 12), (1154, 665, 210), (204, 538, 798), (6, 26, 608) and (0,0, 93) for the ≤40, >40-≤45, >45-≤50, >50-≤55, >55-≤60 and >60 age groups respectively. Due to few observations in each group data not shown for <45 or >55 years.

For all other risk factors, analyses were based on the following number of observations in the (pre, peri, post) groups for the ≤40, >40-≤45, >45-≤50, >50-≤55, >55-≤60 and >60 age groups respectively = (216, 5, 1), (1459, 30, 21), (1980, 1216, 378), (451, 1063, 1570), (12, 73, 1180) and (0,1, 185) for the ≤40, >40-≤45, >45-≤50, >50-≤55, >55-≤60 and >60 age groups, respectively. Due to few observations in each group data not shown for <45 or >55 years.

# Table S8 Mediation analysis of the association between chronological age and CIMT by changes in risk factors

| **Risk factor/ mediator** | **Total effect of age (at clinic assessment 1) on CIMT (at clinic assessment 4) – not via listed risk factor** | | **Direct effect of age (at clinic assessment 1) on CIMT (at clinic assessment 4) – not via listed risk factor** | | **Indirect effect of age (at clinic assessment 1) on CIMT (at clinic assessment 4) – via listed risk factor** | |
| --- | --- | --- | --- | --- | --- | --- |
|  | MD (95% CI) | p-value | MD (95% CI) | p-value | MD (95% CI) | p-value |
| *Cardiovascular measures at clinic 2* | | | | | | |
| Total lean mass (kg/m^2^) | 3.71 (0.82,6.59) | 0.012 | 3.97 (1.08,6.85) | 0.007 | -0.26 (-0.60,0.09) | 0.143 |
| SBP (mmHg) | 3.71 (0.82,6.59) | 0.012 | 3.29 (0.35,6.23) | 0.028 | 0.42 (0.02,0.81) | 0.038 |
| DBP (mmHg) | 3.71 (0.82,6.59) | 0.012 | 3.68 (0.77,6.58) | 0.013 | 0.03 (-0.17,0.23) | 0.785 |
| Pulse rate (bpm) | 3.71 (0.82,6.59) | 0.012 | 3.70 (0.82,6.58) | 0.012 | 0.00 (-0.09,0.10) | 0.950 |
| Triglycerides, logged (mmol/l) | 3.71 (0.82,6.59) | 0.012 | 3.67 (0.77,6.57) | 0.014 | 0.04 (-0.35,0.43) | 0.841 |
| Non-HDL-c (mmol/l) | 3.71 (0.82,6.59) | 0.012 | 4.00 (1.12,6.88) | 0.007 | -0.29 (-0.82,0.25) | 0.288 |
| Glucose, logged (mmol/l) | 3.71 (0.82,6.59) | 0.012 | 3.63 (0.73,6.52) | 0.014 | 0.08 (-0.12,0.28) | 0.428 |
| *Cardiovascular measures at clinic 3* | | | | | | |
| Total lean mass (kg/m^2^) | 3.71 (0.82,6.59) | 0.012 | 3.86 (0.97,6.76) | 0.009 | -0.16 (-0.45,0.14) | 0.302 |
| SBP (mmHg) | 3.71 (0.82,6.59) | 0.012 | 3.42 (0.49,6.35) | 0.023 | 0.29 (-0.07,0.65) | 0.110 |
| DBP (mmHg) | 3.71 (0.82,6.59) | 0.012 | 3.68 (0.80,6.57) | 0.013 | 0.02 (-0.12,0.17) | 0.746 |
| Pulse rate (bpm) | 3.71 (0.82,6.59) | 0.012 | 3.72 (0.83,6.60) | 0.012 | -0.01 (-0.12,0.10) | 0.830 |
| Triglycerides, logged (mmol/l) | 3.71 (0.82,6.59) | 0.012 | 3.34 (0.46,6.22) | 0.023 | 0.37 (-0.08,0.81) | 0.108 |
| Non-HDL-c (mmol/l) | 3.71 (0.82,6.59) | 0.012 | 3.84 (0.96,6.71) | 0.009 | -0.13 (-0.73,0.47) | 0.673 |
| Glucose, logged (mmol/l) | 3.71 (0.82,6.59) | 0.012 | 3.69 (0.81,6.57) | 0.012 | 0.02 (-0.11,0.15) | 0.780 |
| *Difference between clinics 2 and 3* | | | | | | |
| Total lean mass (kg/m^2^) | 3.71 (0.82,6.59) | 0.012 | 3.55 (0.69,6.42) | 0.015 | 0.15 (-0.16,0.47) | 0.336 |
| SBP (mmHg) | 3.71 (0.82,6.59) | 0.012 | 3.69 (0.82,6.57) | 0.012 | 0.01 (-0.11,0.14) | 0.822 |
| DBP (mmHg) | 3.71 (0.82,6.59) | 0.012 | 3.72 (0.83,6.60) | 0.012 | -0.01 (-0.15,0.14) | 0.915 |
| Pulse rate (bpm) | 3.71 (0.82,6.59) | 0.012 | 3.71 (0.81,6.61) | 0.012 | -0.00 (-0.13,0.12) | 0.959 |
| Triglycerides, logged (mmol/l) | 3.71 (0.82,6.59) | 0.012 | 3.65 (0.78,6.53) | 0.013 | 0.06 (-0.21,0.32) | 0.683 |
| Non-HDL-c (mmol/l) | 3.71 (0.82,6.59) | 0.012 | 3.63 (0.74,6.51) | 0.014 | 0.08 (-0.15,0.31) | 0.498 |
| Glucose, logged (mmol/l) | 3.71 (0.82,6.59) | 0.012 | 3.70 (0.82,6.58) | 0.012 | 0.01 (-0.09,0.11) | 0.906 |

The total effect of chronological age (at the first clinic assessment) on CIMT (at the last clinic assessment) was estimated by regressing CIMT on chronological age, adjusted for reproductive age, CIMT (at the first clinic assessment) and baseline confounders. The direct effect (i.e., effect of chronological age on CIMT not via the mediator) was estimated in the same way as the total effect but additionally included the mediator.

# Figure S3 Associations of reproductive and chronological aging with all cardiometabolic measures: sensitivity analyses


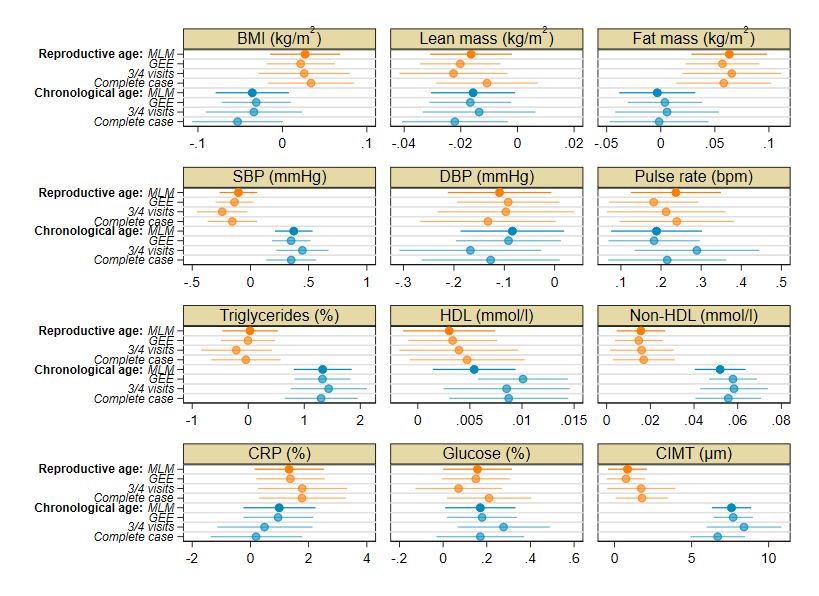


MLM: Multilevel level model; GEE: general estimating equation.

# Figure S4 Associations of reproductive and chronological aging with all cardiometabolic measures: sensitivity analysis excluding women with a plaque


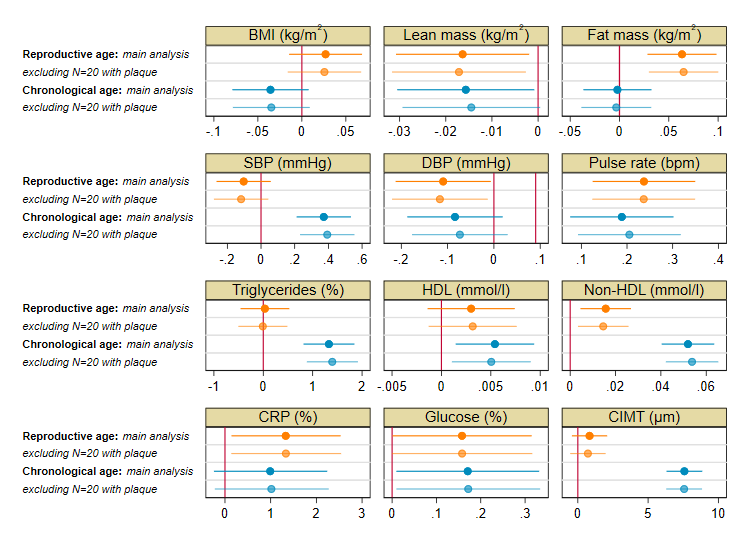

Supplement: Supplementary file 2 — Additional file 2: Table S5. Main analysis: unadjusted and adjusted models. Table S6. Mean difference in outcome by menopausal status. Table S7. Predicted mean differences at ages 45, 50 and 55 by menopausal status. Table S8. Mediation analysis. Figure S1. Shape of non-linear trajectories: fat mass and non-HDL-c. Figure S2. Main analysis: unadjusted and adjusted models. Figure S3. Sensitivity analyses: main model (multilevel) compared to general estimating equation model and sensitivity to number of visits. Figure S4. Sensitivity analyses: excluding women with a plaque. [file 12916_2022_2454_MOESM2_ESM.docx]
