# Supplementary material for: Cardiovascular health in the menopause transition: a longitudinal study of up to 3892 women with up to four repeated measures of risk factors
Source: BMC Med. 2022 Aug 17;20:299. doi: 10.1186/s12916-022-02454-6 (PMC9382827; doi:10.1186/s12916-022-02454-6)
Supplement: Supplementary file 3 — Additional file 3: Table S9. Comparison of age and cardiometabolic risk factors between women included/excluded. Table S10. Comparison of confounders between women included/excluded. [file 12916_2022_2454_MOESM3_ESM.docx]

# Additional file 3

# Contents

- Table S9 Comparison of age and cardiometabolic risk factors between women included (n=1702) and not included (n=2190) in the main analyses
- Table S10 Comparison of confounders between women included (n=1702) and not included (n=2190) in the main analyses

# Table S9 Comparison of age and cardiometabolic risk factors between women included (n=1702) and not included (n=2190) in the main analyses

| Characteristics | 1^st^ assessment | | 2^nd^ assessment | | 3^rd^ assessment | | 4^th^ assessment | |
| --- | --- | --- | --- | --- | --- | --- | --- | --- |
|  | Included | Excluded | Included | Excluded | Included | Excluded | Included | Excluded |
| Age (years) at clinic, mean (SD) | 45.3 (3.4) | 50.5 (4.0) | 47.8 (3.2) | 53.0 (3.9) | 48.9 (3.3) | 54.6 (3.7) | 49.9 (3.2) | 55.7 (3.4) |
| BMI, mean (SD) | 26.8 (5.4) | 25.9 (5.0) | 26.6 (5.4) | 25.7 (4.7) | 26.7 (5.3) | 25.8 (4.9) | 26.7 (5.6) | 25.8 (4.9) |
| TLM, mean (SD) | 15.4 (1.7) | 15.1 (1.5) | 15.7 (1.7) | 15.3 (1.5) | 15.4 (1.6) | 15.0 (1.5) | 15.5 (1.7) | 14.9 (1.6) |
| TFM, mean (SD) | 10.2 (4.1) | 9.6 (3.8) | 9.9 (3.9) | 9.6 (3.4) | 10.1 (4.1) | 9.6 (3.8) | 10.4 (4.1) | 10.1 (3.6) |
| SBP, mean (SD) | 117.5 (11.9) | 118.4 (12.9) | 121.3 (14.3) | 121.3 (14.2) | 118.7 (13.9) | 118.9 (14.1) | 118.5 (14.1) | 119.6 (14.7) |
| DBP, mean (SD) | 71.4 (8.2) | 71.6 (8.2) | 71.9 (10.0) | 71.4 (9.2) | 70.4 (9.4) | 70.0 (8.9) | 70.5 (9.5) | 70.0 (9.3) |
| Pulse rate, mean (SD) | 67.4 (8.7) | 66.6 (8.6) | 70.1 (9.8) | 69.1 (9.5) | 71.6 (10.2) | 70.8 (10.0) | 69.3 (9.9) | 68.6 (9.8) |
| TG, median (IQR) | 0.9 (0.7, 1.2) | 0.9 (0.7, 1.1) | 0.9 (0.7, 1.2) | 0.9 (0.7, 1.2) | 0.8 (0.7, 1.1) | 0.8 (0.7, 1.1) | 0.9 (0.7, 1.2) | 0.9 (0.7, 1.3) |
| Non-HDL, mean (SD) | 3.4 (0.9) | 3.5 (0.9) | 3.4 (0.7) | 3.7 (0.9) | 3.4 (0.8) | 3.7 (0.9) | 3.7 (0.8) | 4.0 (0.9) |
| HDL, mean (SD) | 1.4 (0.4) | 1.6 (0.4) | 1.5 (0.3) | 1.6 (0.3) | 1.6 (0.3) | 1.6 (0.3) | 1.6 (0.3) | 1.6 (0.4) |
| CRP, median (IQR) | 0.9 (0.5, 2.1) | 0.9 (0.4, 1.9) | 1.1 (0.5, 2.4) | 1.0 (0.5, 2.1) | 1.0 (0.5, 2.3) | 1.0 (0.5, 2.2) | 1.1 (0.6, 2.4) | 1.1 (0.5, 2.4) |
| Glucose, median (IQR) | 5.2 (4.9, 5.4) | 5.2 (4.9, 5.5) | 5.2 (4.9, 5.4) | 5.2 (5.0, 5.5) | 5.1 (4.9, 5.4) | 5.2 (4.9, 5.5) | 5.3 (5.0, 5.5) | 5.3 (5.1, 5.6) |
| CIMT, median (IQR) | 0.55 (0.51, 0.58) | 0.57 (0.53, 0.60) | NA | NA | NA | NA | 0.58 (0.53, 0.65) | 0.62 (0.56, 0.69) |
|  |  |  |  |  |  |  |  |  |

# Table S10 Comparison of confounders between women included (n=1702) and not included (n=2190) in the main analyses

| **Confounders** |  | Included | Excluded |
| --- | --- | --- | --- |
| BMI at first assessment | Normal | 1287/1496 (86.0) | 1579/1915 (82.5) |
|  | Overweight | 170/1496 (11.4) | 261/1915 (13.6) |
|  | Obese | 39/1496 (2.6) | 75/1915 (3.9) |
| Smoking status | Never smoker | 809/1483 (54.6) | 1044/1849 (56.5) |
|  | Former smoker | 525/1483 (35.4) | 587/1849 (31.7) |
|  | Current smoker | 149/1483 (10.0) | 218/1849 (11.8) |
| Alcohol intake frequency | Never or less than 4 times a month | 468/1155 (40.5) | 592/1317 (45.0) |
|  | 2 to 3 times a week | 384/1155 (33.2) | 457/1317 (34.7) |
|  | 4 or more times a week | 303/1155 (26.2) | 268/1317 (20.3) |
| Parity | 1 | 194/1702 (11.4) | 255/2190 (11.6) |
|  | 2 | 567/1702 (33.3) | 733/2190 (33.5) |
|  | 3 | 423/1702 (24.9) | 582/2190 (26.6) |
|  | 4+ | 518/1702 (30.4) | 620/2190 (28.3) |
| Age at menarche | Early (≤ 11 years) | 242/1507 (16.1) | 351/1958 (17.9) |
|  | Average (12-14 years) | 1055/1507 (70.0) | 1368/1958 (69.9) |
|  | Late (≥ 15 years) | 210/1507 (13.9) | 239/1958 (12.2) |
| Educational achievement | CSE / Vocational degree/ O-level | 688/1587 (43.4) | 1180/2032 (58.1) |
|  | A-level | 498/1587 (31.4) | 535/2032 (26.3) |
|  | University degree | 401/1587 (25.3) | 317/2032 (15.6) |
