# Supplementary material for: Cardiovascular health in the menopause transition: a longitudinal study of up to 3892 women with up to four repeated measures of risk factors
Source: BMC Med. 2022 Aug 17;20:299. doi: 10.1186/s12916-022-02454-6 (PMC9382827; doi:10.1186/s12916-022-02454-6)
Supplement: Supplementary file 4 — Additional file 4: Table S11. Summary of relevant longitudinal studies. [file 12916_2022_2454_MOESM4_ESM.docx]

# Additional file 4

# Contents

- Table S11 Summary of longitudinal studies that assessed the association of cardiovascular risk factors with reproductive and/or chronological age

# Table S11 Summary of longitudinal studies that assessed the association of cardiovascular risk factors with reproductive and/or chronological age

|  | Author, year  Country | Sample characteristics | Outcomes | Follow-up and repeat measures | Exposure | Covariates | Results |
| --- | --- | --- | --- | --- | --- | --- | --- |
| 1 | Matthews 2009  USA,  Sub study of SWAN (12) | 1,054 women had achieved an FMP not due to surgery and without hormone therapy use before FMP. | Lipids, CRP, glucose, blood pressure | 10 annual examinations | FMP, age | ethnicity; site;  baseline height; baseline log weight and change in log  weight; concurrent smoking, concurrent  relevant medication use (antihypertensive use for  analyses of blood pressure and pulse pressure, insulin use  for analyses of glucose and insulin, lipid-lowering medications for lipids. | - LDL demonstrated substantial increases within the 1-year interval before and after the FMP, consistent with menopause-induced changes.  - HDL-c increase until 1yr post menopause then gradually decreases.  - The analyses showed no influence of the FMP on blood pressure, and, glucose. |
| 2 | Derby 2009 USA  Sub study of SWAN (8) | 2,659 women | Lipids | Up to 7 observations (average, 3.9). | FMP, age | Age, BMI, smoking, medical history, and medication use. | - Serum lipids increased modestly during the menopause transition, peaking during late peri- and early postmenopause.  - HDL-c increased gradually between pre and late perimenopause but then a decrease postmenopause. |
| 3 | Do 2000  Australia (36)  Melbourne Women’s midlife health project | From 1991 to 1995 of 150 middle-aged Melbourne, Australia, women as they passed through menopause. | HDL,  LDL, triglycerides, DBP and BMI | 4 year follow-up from 1991 to 1995 with up to 4 repeat measures | FMP. age | Smoking, alcohol, exercise | - net increases in HDL (0.05 mmol/litre for), LDL (0.25 mmol/litre), triglycerides (0.34 mmol/litre), DBP (48 mmHg) and BMI (0.12 kg/m2) between 3 years before and 3 years after the menopause. |
| 4 | Greendale 2019  Swan sub-study (15) | 1246 participants, Mean baseline age was 47.1 years (SD, 2.6 years) and average age at FMP was 52.2 years (SD, 2.8 years). | Fat mass, lean mass | 8 years  before through 10.5 years after the FMP. Median number of visits was 10. | FMP, age | Ethnicity, study site, and hormone therapy. | Fat and lean mass increased prior to the MT. At the start of the MT, rate of fat gain doubled, and lean mass declined; gains and losses continued until 2 years after the FMP. E.g., Menopause transition: 2 years before to 1.5 years after FMP for fat mass: 2.11%  (1.65%, 2.57%) and lean mass –0.21%  (–0.37%, –0.04%). |
| 5 | El Khoudary  Sub study of SWAN (18) | 249 Women (42–57 years old, premenopausal (49%) or early peri-menopausal  (46%)) were included. | CIMT | Followed up for up to 9 years (median=3.7 years) and had up to 5 carotid  scans. | Menopausal stages, age | Ethnicity | The overall rate of change in IMT was 0.007 mm/year. Independent of age and race,  progression rate of IMT increased substantially in late peri-menopausal stage (0.017 mm/year)  compared to both premenopausal (0.007 mm/year) and early peri-menopausal (0.005 mm/year)  stages; (P≤0.05). |
| 6 | Janssen  Sub study of SWAN (33) | 949 women | HDL-c  Triglycerides  SBP  Glucose | 9 year follow-up period. | Age, Age at FMP | Ethnicity, study  site, marital status, education, smoking, BMI at baseline, and  change in BMI from baseline. | HDL-C and triglycerides increased significantly during the perimenopausal period but stabilized afterward.  Glucose level decreased slightly but significantly with aging during the entire study period.  SBP increased. |
| 7 | Matthews 2021  Sub study of SWAN (16) | 1554 pre menopausal women who had a natural menopause during. 890 women also had measures CIMT.  Mean age at baseline:  46.41 (2.70). | CIMT  Triglycerides  HDL-c, non-HDL-c | Follow-up years (median=18.8 years). | Age, Age at FMP | Adjustment for site, race/ethnicity, education, and age at baseline  (model 1), and model 1 covariates plus time varying  lipid lowering medications, CVD events, smoking status,  body mass index, and HT (model 2). | Triglycerides significant increases until 3 years post-FMP and then declined. Similarly for Non-HDL-c. HDL-c increased.  CIMT was associated with older age at FMP. |
| 8 | Guthrie 2001 (37)  Melbourne Women’s midlife health project | A total of 265 women (110 pre-, 138 peri-, 17 postmenopausal) aged 46–57. | Glucose, insulin | 5 years annually | FMP, age |  | The onset of impaired fasting glycaemia was not triggered by the menopausal transition or hormone use. Changes in insulin concentration were associated with changes in BMI (P<0.05). |
| 9 | Matthews 2013  Sub study of SWAN (35) | 1,769 women had reached natural menopause | HDL-c, LDL-c, SBP, CRP | 11 years, annually | Natural menopause vs hysterectomy with ovarian conservation vs hysterectomy with bilateral oophorectomy | Models adjusted by site, race/ethnicity, educational attainment, age at FMP or surgery, menopausal status at visit before FMP or surgery, elapsed time between index visit and FMP or surgery (all time-invariant), and stroke or MI, physical activity, antidepressant use (HDL, triglycerides), HRT, cholesterol medication use and smoking | SBP: mean (SE) p-value: 0. 01 (0.10) p=0.92; CRP: 0.14 (0.08) p=0.07;  HDL-c: 0.82 (0.15) <0.001; non-HDL-c: 1.52 (0.37) <0.001; log trigs: 0.01 (0.00) <0.001 |
| 10 | Appiah (34) The CARDIA Study | n = 588 women who had a natural menopause | HDL-c, LDL-c, SBP, fasting glucose, BMI | 25 years of follow-up | Natural menopause vs hysterectomy with ovarian conservation vs hysterectomy with bilateral oophorectomy | for time-invariant covariates (race, study center, educational level, baseline body mass index (weight (kg)/height(m)2), age at final menstrual period or surgery, and elapsed time between FMP or surgery and the index visit (the visit after final menstrual period or surgery)) and time-varying covariates (smoking status, physical activity level, parity, diabetes, and use of oral contraceptives, HRT, or cholesterol-lowering medications) | mean (SE):Non-HDL: 1.13 (0.39) HDL-c: 0.69 (0.19); log trigs 0.007 (0.01)  BMI: 0.22 (0.06); SBP: 0.47 (0.19); Fasting glucose: 0.01 (0.28) |

SWAN: Study of Women's Health Across the Nation
